# Pathogen-induced damage in *Drosophila*: Uncoupling disease tolerance from resistance

**DOI:** 10.1371/journal.ppat.1013482

**Published:** 2025-09-19

**Authors:** Priscilla A. Akyaw, Tânia F. Paulo, Elvira Lafuente, Élio Sucena

**Affiliations:** 1 Instituto Gulbenkian de Ciência, Oeiras, Portugal; 2 GIMM - Gulbenkian Institute for Molecular Medicine, Lisbon, Portugal; 3 Departamento de Biologia Animal, Faculdade de Ciências, Universidade de Lisboa, Lisbon, Portugal; 4 cE3c: Centre for Ecology, Evolution and Environmental Changes, Faculdade de Ciências, Universidade de Lisboa, Lisbon, Portugal; 5 CHANGE – Global Change and Sustainability Institute, University of Lisbon, Lisbon, Portugal; University of Kansas, UNITED STATES OF AMERICA

## Abstract

Immune response against infections can be divided into mechanisms of resistance that ensure active pathogen elimination, and mechanisms of disease tolerance, which include processes that return the host to physiological homeostasis without direct control of pathogen load. Studies on host immune response to infection have targeted mechanisms of resistance, and consequently, these are now well-described in both vertebrates and invertebrates. By comparison, the mechanistic basis of disease tolerance is poorly understood. This is in part because both processes interact and can be difficult to disentangle under an infection scenario. Using the insect model *Drosophila melanogaster* exposed to its natural entomopathogen, *Pseudomonas entomophila*, we aimed to tease apart mechanisms of disease tolerance from those of resistance. To this end, we reasoned that the response to oral exposure to heat-killed entomopathogenic bacteria, whilst initially triggering both resistance and disease tolerance mechanisms, would be resolved mainly by disease tolerance alone. Using this method, we observe that oral exposure to heat-killed *P. entomophila* causes mortality and reduced fecundity in *D. melanogaster*. We confirm that this reduction in fitness-related traits depends on the duration of the exposure, is sexually dimorphic, and is dependent on the virulence of the bacterium. We also found the microbiota to play a role, with its presence exacerbating the deleterious effect on host survival. In addition, we show that the Imd pathway, but not effector genes, is involved in the process of surviving exposure to HK bacteria. This experimental framework, which may be extended to other systems, can be instrumental towards an understanding of the molecular, genetic, and physiological basis of disease tolerance and its interactions with resistance mechanisms.

## Author summary

Host immune response against infections can be divided into mechanisms of resistance that ensure active pathogen elimination, and mechanisms of disease tolerance, which include processes that return the host to physiological homeostasis without involving direct pathogen control. Using a protocol that exposes *Drosophila melanogaster* to an inactivated form of *Pseudomonas entomophila*, we aimed to assess mechanisms of disease tolerance separately from those of resistance and revealed measurable pathogen-induced damage and host fitness costs (survival and fecundity). We show that this reduction in fitness-related traits increases with the duration of the exposure, is dependent on bacterial virulence factors, and is sexually dimorphic, with the microbiota exacerbating deleterious effect on host survival.

This methodological framework provides a valuable experimental tool to isolate disease tolerance from resistance, offering deeper insights into how hosts mitigate fitness costs associated with bacterial infections. Extension to other systems can be instrumental towards a generalized understanding of the molecular, genetic, and physiological bases of disease tolerance and its interactions with resistance mechanisms.

## Introduction

The ability to overcome an infection without enduring deleterious fitness consequences strongly influences the evolution of both hosts and pathogens [1,2]. Hosts employ a variety of countermeasures against pathogens including behavioural avoidance (e.g., avoiding contaminated food, water, and other infected hosts) [3–6], and the classical immunological responses involving mechanisms of resistance (i.e., the use of immune effectors to eliminate or reduce pathogen load) and of disease tolerance (i.e., the minimizing of the effects of damage caused by the infection process) [7–10]. These strategies ultimately ensure the prevention and control of infection to promote host survival and reproduction [10–12].

In the last two decades, *D. melanogaster* has been instrumental in further characterizing these two arms of the immune response [13–21]. A pivotal moment was the discovery of the involvement of the Toll and IMD pathways in the mechanisms of resistance that target invading pathogens for elimination, through the regulation of antimicrobial peptides (AMPs) and reactive oxygen species (ROS) production [18,22,23]. These and subsequent studies have uncovered the functional roles of specific AMPs such as Cecropin (Cec) [24–26], Diptericin (Dpt) [22,27,28], Defensin (Def) [29,30] and Drosomycin (Drs) [31–33], as well as the dynamics of their transcriptional activation during infection. More recent research has shown how these processes lead to pathogen clearance [22,34] and/or pathogen-induced damage control [35–37], as well as provided insights into the regulatory mechanisms that govern them [13,38–40].

In contrast, disease tolerance still awaits a comparably deep mechanistic characterization. It is expected that the processes involved include the maintenance of homeostatic conditions and normal physiological functions by repair and/or prevention of damage caused during infection [41–45]. Operationally, disease tolerance is measured as the relative status of a health criterion upon infection or of a proxy measure of immune elicitors/effectors [10,41,46–50]. However, even the seemingly simple choice of what criterion should be considered and what parameters measured to assess physiological state, is highly variable and encompasses distinct phenotypes and terminology that include “fitness” [11,44,51], “health”, and “performance” [10,49,50,52]. Moreover, resistance and disease tolerance are likely to be mechanistically and evolutionarily intertwined [10,11,17,46,48,53–56]. Ultimately, the knowledge gap regarding the mechanisms of disease tolerance is partially explained by the difficulty in disentangling them from resistance in a solid operational way, allowing for focused experimental approaches [10,50,51,53,57].

One first step in unravelling the mechanisms underlying disease tolerance has been to distinguish mortality tolerance, defined as higher survival under comparable pathogen loads, from fecundity tolerance, understood as maintaining reproductive output during infection [11,58,59]. Studies focusing on mortality tolerance have uncovered a number of genes, including *Eiger*, *CrebA*, *Pirk*, and *IRC*, involved in promoting host survival upon systemic infection without affecting pathogen load [60–63]. Yet, the specific ways in which these genes shape this response and the nature of the physiological processes in which they participate, are not fully understood.

To gain a deeper understanding of disease tolerance, it is fundamental to characterize the consequences of infection on other important life-history traits, such as reproduction, stress response, growth, tissue repair, as well as energy acquisition and allocation [46,53,58,64–67]. Furthermore, it is also critical to understand whether such processes and mechanisms promoting disease tolerance are general or deployed differently, according to variations in challenge such as distinct pathogens or routes of infection [68,69]. Notwithstanding, disease tolerance also encompasses *sensu latu* response to damage, whether directly inflicted by the pathogen or self-inflicted [39,41,44,54,60,70].

In insects, there is evidence that immunopathology contributes to mortality during infection [39,48,60,71–73]. In particular, previous work in *D. melanogaster* established that immune activation is achieved by the recognition of microbial cell wall components and by virulence factors secreted by specific pathogens [60,74–76]. Therefore, *D. melanogaster* is likely to sustain these two non-mutually exclusive types of fitness costs when it is infected: indirect damage induced by immunopathology and direct damage imposed by the pathogen (via secretion of virulence factors and toxins). Responding to either or both types of damage requires mechanisms of disease tolerance.

To understand how disease tolerance promotes host fitness independently from the action of resistance in reducing pathogen load, we measured survival and fecundity of *D. melanogaster* after exposure to an inactivated (heat-killed) form of *Pseudomonas entomophila*. This gram-negative bacterium is a natural entomopathogen of *D. melanogaster* that, ensuing the detection of virulence factors and/or microbial cell wall components, activates both local and systemic immune responses at different life stages and contexts [75–77]. Although *Drosophila* deploys a very strong immune response upon oral infection, mainly through the upregulation of specific AMPs and ROS towards this bacterium [78], it suffers severe damage to the gut epithelium, leading to high mortality within the first 24 hours after infection [62,79,80].

In this work, we measured the mortality and fecundity tolerance exhibited by *D. melanogaster* when fed with heat-killed *P. entomophila*. We reasoned that the host response to oral exposure to heat-killed pathogenic bacteria should trigger both disease tolerance and resistance responses (i.e., AMP production). However, in the absence of a proliferating pathogen to control, resolving this challenge should mostly rely on disease tolerance mechanisms. We first characterized how feeding on heat-killed bacteria affected host survival and fecundity, and quantified damage to the gut epithelium after this treatment. Additionally, we measured gene expression profiles of key components of the *Drosophila* immune response, such as canonical AMPs, and components of the ROS and stress response pathways. In this way, we have established a new experimental framework with which to measure disease tolerance to pathogen-derived damage that allows for the disentangling of these effects from their immune-resistance counterparts. With this approach, we hope to contribute to coming one step closer towards the mechanistic basis for disease tolerance in an oral infection context.

## Results

### 1. Establishing a protocol to measure disease tolerance

**Fig 1:**
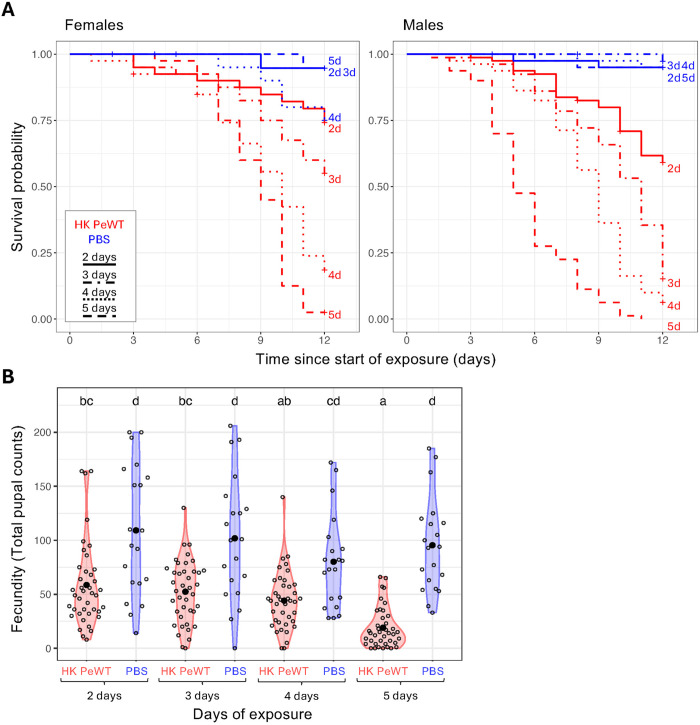
Exposure to heat-killed *P. entomophila* entails survival and fecundity costs. Survival and fecundity were monitored for 12 days in flies exposed to heat-killed (HK) *P. entomophila* (HK PeWT - red) or control food (PBS - blue). A) Survival curves for females (left panel) and males (right panel) exposed for two, three, four, or five days. HK *P. entomophila* treatment led to increased mortality as compared to PBS, independently of the number of days of exposure (p < 0.05 for all comparisons), except for females in the two-day exposure group. B) Female reproductive output measured as total pupal counts. Flies exposed to HK *P. entomophila* have consistently lower reproductive output compared to control, with the 5-day exposure regime showing a significantly stronger effect than the other exposure times. Differences between groups were estimated by post hoc comparisons (Tukey’s honest significant differences) and are indicated by different letters in each plot (p < 0.05).

The gram-negative bacterium *Pseudomonas entomophila* is highly virulent to *Drosophila melanogaster*, killing more than 50% of flies within 48 hours after ingestion [75,81]. To determine whether oral exposure to food containing heat-killed (HK) *P. entomophila*, would induce a measurable fitness cost in *D. melanogaster*, we fed it to adults for periods of two, three, four, or five days after which, we measured survival (Fig 1A) and fecundity (Fig 1B) for 12 days. We established a heat inactivation-based protocol using 55ºC (S1 Fig) as this was the lowest temperature at which we did not observe bacterial colonies upon subsequent plating (S2A Fig).

We observed a significant effect of treatment on mortality that was dependent of sex (Anova(Cox): χ²(1)= 8.9, p = 0.003) and length of exposure (Anova(Cox): χ²(3) = 10.13, p = 0.02) (Fig 1A, S1, S2 Tables). Except for females from the two-day exposure treatment (EMMs(Cox): est = -1.74 (SE = 1.05), p = 0.097), all flies exposed to food mixed with HK *P. entomophila* were more susceptible than those exposed to food mixed with PBS (p < 0.05 in all cases), and the longer the exposure, the highest the mortality (Fig 1A, S2 Table). Flies exposed for two days had the highest survival rates, with 74% for females and 59% for males. The three-day exposure group showed a steeper survival decline: females showed 55% survival and males only 15%. The five- day exposure resulted in the lowest survival, with only 2% for females and no surviving males by day 12. There was a significant difference between the number of exposure days for all paired combinations (p < 0.01 in all cases) except for females exposed for two and three days (EMMs(Cox): est = -0.69 (SE = 0.39), p = 0.3) and between four and five days (EMMs(Cox): est = -0.54 (SE = 0.25), p = 0.13) (Fig 1A, S2 Table). These results also reveal a strong sexual dimorphism in the response to oral exposure to HK *P. entomophila*, with males having a significantly higher risk of death (approximately twice) compared to females (Cox: HR = 2.12, p < 0.001) (S2B Table). With regards to fecundity, we also found it to be significantly reduced in flies exposed to HK *P. entomophila* as compared to the control group (Anova(lmer): χ²(1) = 23.21, p = 1.45e-06) (Fig 1B), with a tendency to correlate negatively with the length of exposure, though only significant when comparing four and five days to two and three days exposures (Fig 1B, S2 Table). The effect of exposure to HK *P. entomophila* led to an average reduction in fecundity of about 55% relative to PBS control (i.e., mean fecundity estimates were 96.6 and 43.5 for the HK and the PBS treatments, respectively) (Fig 1B, S2 Table).

Having shown that feeding heat-killed *P. entomophila* negatively impacted both survival and fecundity in adult flies, we sought to generalize this effect by testing its independence from the bacteria inactivation method itself. To that aim, we first inactivated the bacteria with an alternative method consisting of incubation in paraformaldehyde (PFA) (S3A Fig). PFA inactivation affected host survival negatively (S1 Table) compared to the control group (EMMs(Cox): est = -3.11 (SE = 0.60), p = 1.15e-06 for females and est = -2.01 (SE = 0.30), p = 2.51e-11 in males), showing a similar tendency to that of the heat-killing protocol for both sexes (S3A Fig, S3 Table). Secondly, to gain insight into the relationship between the observed effects on survival and the level of denaturation of the bacteria, we tested a harsher heat inactivation temperature of 95ºC. Analyses of survival upon exposure to the two heat-killing temperatures, revealed a significant effect of this variable (S1 Table) most notably in females (i.e., significant temperature by sex interaction) (Anova(Cox): χ²(2) = 26.8, p = 1.54e-06), where inactivation at 95 ºC led to considerably more mortality than inactivation at 55 ºC (S3B Fig, S1 and S3 Tables).

In conclusion, exposure to inactivated *P. entomophila* led to increased mortality and reduced fecundity (shown for heat-inactivated bacteria), both scaling positively with the duration of exposure. Based on these findings, all subsequent experiments were performed under a protocol using a three-day exposure to 55ºC heat killed *P. entomophila*.

### 2. Mortality after exposure to heat-killed bacteria depends on its pathogenicity

To determine whether the fitness costs observed in flies after exposure to heat-killed bacteria were specific to the entomopathogen *P. entomophila* or if they reflected a general response, we tested additional bacterial species with the same protocol. For that, we followed survival of flies fed with four different HK gram-negative bacteria with described varying levels of pathogenicity through the oral route. We used *Pseudomonas putida*, an avirulent bacterium closely related to *P. entomophila* [82], *Pectobacterium carotovorum* (formerly known as *Erwinia carotovora carotovora*) (strain Ecc-15) which is known to secrete virulence factors but does not induce high mortality in *Drosophila* [83,84] a mutant version of this bacterium in which the virulence factors have been deleted (strain Ecc-71), and *Escherichia coli* (*E. coli*) K-12 strain which is considered non-pathogenic to *D. melanogaster* [85,86].

**Fig 2:**
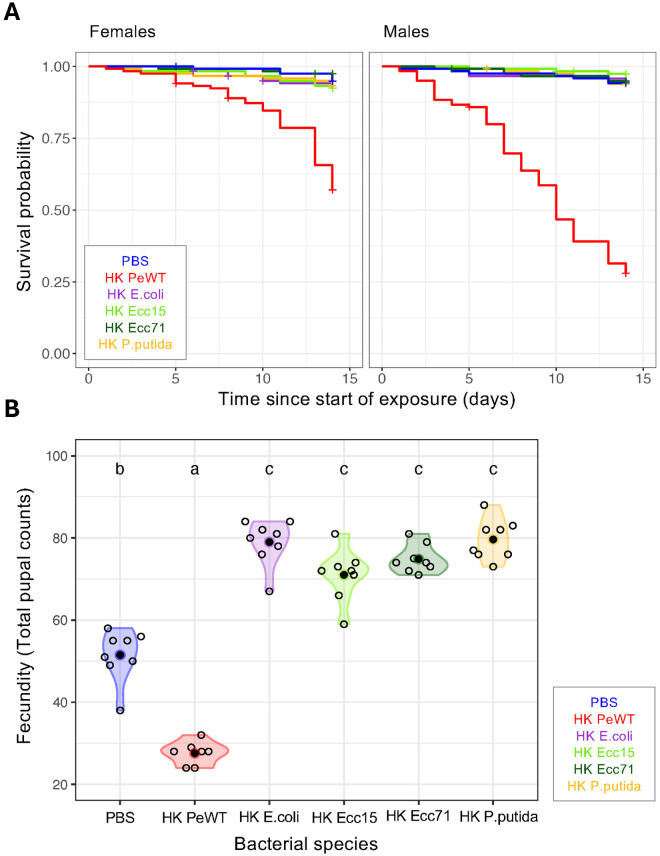
Survival and fecundity upon exposure to heat-killed (HK) bacteria depend on bacterial virulence. Survival and fecundity of flies exposed to PBS control food (PBS - blue) or to heat-killed (HK) bacteria with different levels of virulence: *P. entomophila* (PeWT - red), *Escherichia coli* (E. coli - purple), *Pectobacterium carotovorum* 15 (Ecc15 - light green), *Pectobacterium carotovorum* 71 (Ecc71 - dark green) or *Pseudomonas putida* (*P. putida* - yellow). A) Survival curves in females (left panel) and males (right panel) over 14 days shows that there is no difference in survival between the PBS-exposed group and all other bacteria exposed flies, except for *P. entomophila* (p < 0.05 in all cases). B) Mortality-corrected reproductive output measured as cumulative daily pupal count over 14 days (see Materials and Methods). Reproductive output is lowest upon exposure to HK *P. entomophila*, followed by PBS. Exposure to all other bacteria species leads to increased reproductive output (p < 0.05 in all cases). Differences between groups were estimated by post hoc comparisons (Tukey’s honest significant differences) and are indicated by different letters in each plot (p < 0.05).

Host survival was not significantly affected upon exposure to these heat-killed bacterial species for three days except for *P. entomophila*, which led to higher mortality than that of the PBS control (p > 0.05 for all comparisons) (Fig 2A, S1 and S4 Tables) in a sex-dependent manner (Anova(Cox): χ²(5) = 16.01, p = 0.007).

Concerning female fecundity, we found it to be significantly affected by exposure to the different bacterial species (Anova(glmm): χ²(5) = 256.34, p < 2.2e-16, S1 Table) and different from the PBS control (p < 0.05 in all cases, S4 Table). Notably, all females exposed to non-virulent bacteria showed a higher fecundity, equivalent amongst them, than those fed with PBS (p < 0.05 for all pairwise comparisons, S4 Table). As expected, and previously shown, females exposed to the HK *P. entomophila* displayed a significant decrease in fecundity as compared to control (EMMs(glmm): est = -0.66 (SE = 0.08), p = 1.45e-13) (Fig 2B, S4 Table).

### 3. Virulence factors of heat-killed *P. entomophila* are necessary to induce mortality and fecundity costs

To confirm that the virulence factors of *P. entomophila* cause fitness costs under the HK exposure treatment, we measured survival and fecundity after feeding flies with either wild-type bacteria (hereafter, PeWT) or the mutant avirulent strain *P. entomophila* ΔGacA (hereafter, PeGacA), which carries a Tn5 mini transposon in the GacA gene [80], a part of the GacS/GacA two-component system controlling virulence in *P*. *entomophila* [81].

**Fig 3:**
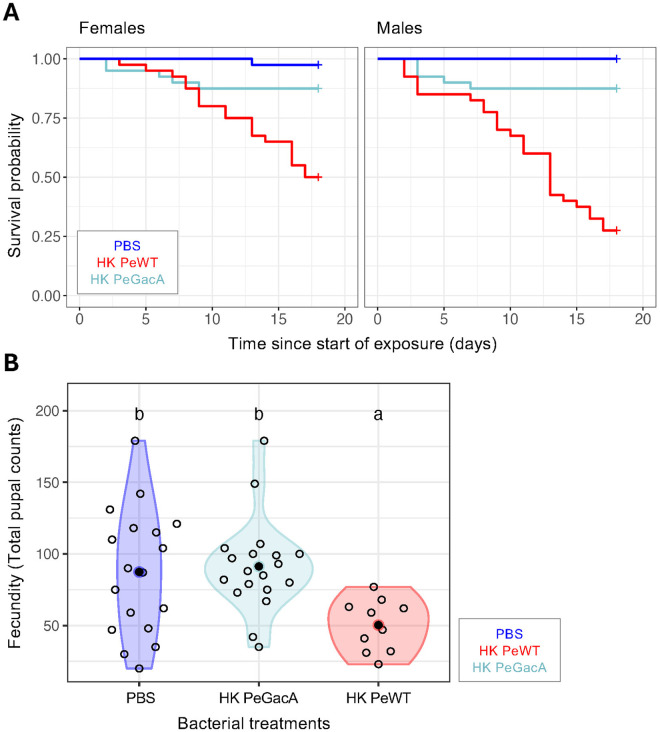
Exposure to a heat-killed non-virulent strain of *P. entomophila* does not affect mortality nor fecundity. Survival and fecundity in flies exposed to food containing heat-killed wild- type *P. entomophila* (PeWT - red), the avirulent *P. entomophila* ΔGacA mutant (PeGacA - light blue), or PBS for control (PBS - dark blue). A) Survival curves of females (left panel) and males (right panel) over 18 days show no difference in survival between the PBS- and GacA-exposed groups (p > 0.05 in both sexes), in contrast with WT-exposed flies which are significantly different for all comparisons (p < 0.01). As previously shown, response to WT *P. entomophila* differed between sexes (p < 0.01). B) Mortality-corrected reproductive output measured as cumulative daily pupal count (see Materials and Methods) shows that reproductive output in HK *P. entomophila* ΔGacA- and PBS-exposed flies do not differ and are both higher than in HK *P. entomophila* WT exposed flies. Differences between groups were estimated by post hoc comparisons (Tukey’s honest significant differences) and are indicated by different letters in each plot (p < 0.05).

In contrast to HK PeWT, exposure to HK PeGacA treatment did not induce high mortality, in neither males nor females (Fig 3A, S1 Table). The PeGacA strain of the bacterium also failed to induce higher mortality than the PBS control in either sex (EMMs(Cox): p > 0.05) (Fig 3A, S5 Table). Once more, we observed strong effects of exposure to HK PeWT on survival, whereby both sexes die more than in the PBS control (Fig 3A, S1 and S5 Tables), more prominently in males (EMMs(Cox): est = 4.18 (SE = 1.1), p = 0.0004) than in females (EMMs(Cox): est = 3.2 (SE = 1.1), p = 0.01), (S5 Table). We extended this experiment to 17 days to evaluate potential delayed effects; however, the survival curves showed relatively steady slopes over the second and third weeks, and therefore, subsequent experiments were conducted for 14 days.

We also observed differences in fecundity between bacterial strains (Anova(glmm): χ²(2) = 32.65, p = 8.15e-08). Flies exposed to HK PeGacA did not show a reduction in fecundity as the one observed with HK PeWT. In fact, fecundity in flies exposed to HK PeGacA was similar to that of flies exposed to PBS control and higher than under exposure to HK PeWT (Fig 3B, S5 Table).

In parallel, and as a somewhat positive control and confirmation of bacteria pathogenicity, we ran the same experiments described above with live *P. entomophila* (S4 Fig, S1 and S6 Tables). Live Infection with the wild-type *P. entomophila* led to mortality of over 90% within five to six days of infection (S4A Fig), as previously reported [17,75,81,87]. We found a significant difference in survival between live PeWT, but not PeGacA, and sucrose treatments (Anova(Cox): χ²(2) = 162.6, p = 5.02e-36) and between sexes (Anova(Cox): χ²(1) = 4.47, p = 3.46e-02) (S4A Fig, S1 and S6 Tables). Fecundity differed between treatments (Anova(glmm): χ²(2) = 157.18, p = 7.40e-35; S1 Table), with the highest fecundity observed in the presence of live GacA, followed by the sucrose control, and the lowest fecundity being displayed upon exposure to live PeWT (S4B Fig, S1 and S6 Tables).

### 4. Exposure to heat-killed *P. entomophila* causes gut damage

**Fig 4:**
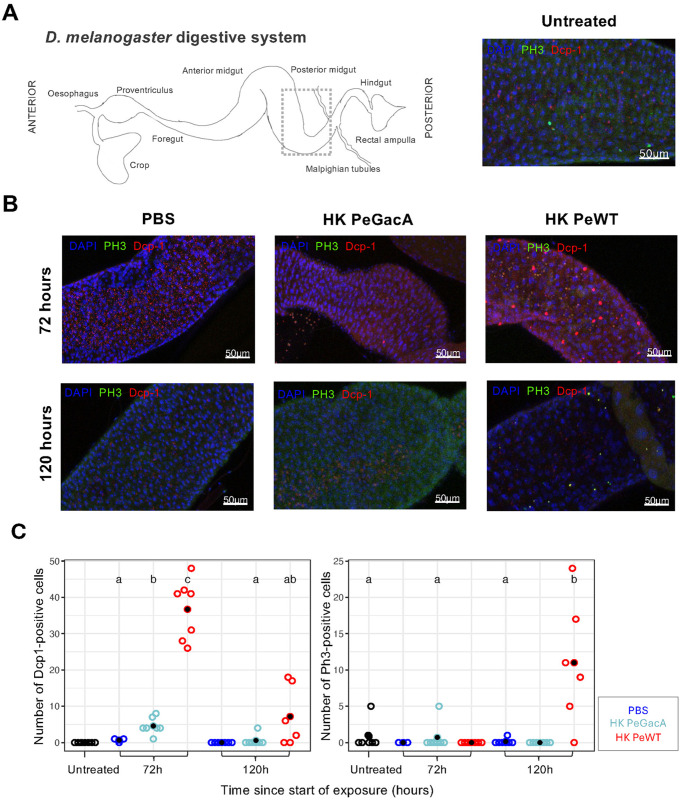
Feeding on heat-killed *P. entomophila* increases apoptosis and mitotic cell division in gut epithelial tissues. A) On the left, a cartoon depicting the *Drosophila* digestive system. The dashed inset box delimits the midgut region shown in subsequent images of immunofluorescent staining (A and B). Immunostaining shows antibodies against *Drosophila* caspase 1 (Dcp-1) in red and Phospho-Histone H3 (PH3) in green, merged with DAPI in blue, as exemplified on the right panel by a picture from a control “Untreated” sample. B) Guts of flies exposed to PBS, heat-killed ΔGacA (HK PeGacA), or heat-killed wildtype (HK PeWT) *P. entomophila* at 72 hours (top) and 120 hours (bottom) after exposure. C) Quantification of Dcp-1 or PH3- positive cells in posterior midgut of males (N = 6 to 8). Exposure to HK PeGacA provoked a significant increase in apoptotic cells at 72 hours (p < 0.05), which dissipated 2 days later. Feeding on HK PeWT led to a large increase in the number of Dcp-1-positive cells (p < 0.01), which remained tendentially elevated at 120 hours. Mitotic gut cell numbers were significantly increased with exposure to HK PeWT at 120 hours (p < 0.01), with no detectable effect on flies fed with HK PeGacA-supplemented food (p > 0.05). Differences between groups were estimated by post hoc comparisons (Tukey’s honest significant differences) and are indicated by different letters in each plot (p < 0.05). Note that statistical group letters are not shown for some groups due to lack of within-group variation, which prevented reliable estimation of contrasts.

The virulence factors of *P. entomophila* are known to induce severe gut epithelium damage during an oral infection [75,76,88]. Having found that exposure to heat-killed *P. entomophila* caused reduced survival and fecundity in a virulence factor-dependent manner (Figs 1–3), we asked if this effect could be downstream of damage inflicted to the gut, as described for infections with live bacteria [79,80,83]. To get a direct measure of cell damage to the posterior midgut region of individuals exposed to HK PeWT, HK PeGacA, or PBS, we quantified the number of apoptotic and mitotic cells using immunohistochemistry staining against genes *Dcp-1* and *PH3*, respectively [89,90] (Fig 4A).

We dissected and stained the guts of three to five-day-old males at three timepoints: before exposure (Untreated), at the end of exposure (72 hours), and 48 hours after transfer to normal food (120 hours) (Fig 4B). Quantification of number of positive cells for apoptotic marker Dcp-1 revealed that exposure to HK PeGacA is sufficient to induce an increase in apoptosis at 72 hours (EMMs(zinf): est = -3.9 (SE = 1.0), p = 0.003), which resolves after removal from the treatment food and into clean food 48 hours later (EMMs(zinf): est = -0.6 (SE = 0.7), p = 1) (Figs 4C and S5, S1 and S7 Tables). Additionally, quantification of the number of positive cells for mitotic marker PH3 detected no significant differences at either timepoint for this treatment (Figs 4C, S5, S7 Table). However, exposure to HK PeWT led to a major increase in the number of apoptotic cells at 72 hours of feeding (EMMs(zinf): est = -36.0 (SE = 4.2), p = 6.5e-14). This number remained elevated for 48 hours after treatment removal and exposure to clean food, although the effect was not statistically significant (EMMs(zinf): est = -7.2 (SE = 2.5), p = 0.06). This trend supports the deleterious effect of HK PeWT on gut-cell integrity. In line with this, we observed a significant increase in the number of dividing cells (EMMs(zinf): est = -10.9 (SE = 3.1), p = 0.01) for HK PeWT guts at 120 hours, suggesting a process of gut health restoration and cell renewal in flies recovering from damage (Figs 4C and S5, S7 Table).

Together, these results show that exposure to heat-killed *P. entomophila* causes tissue damage and underscores the role of tissue repair response against the action of bacterial virulence factors.

### 5. The effects of exposure to heat-killed bacteria are influenced by the microbiota

**Fig 5:**
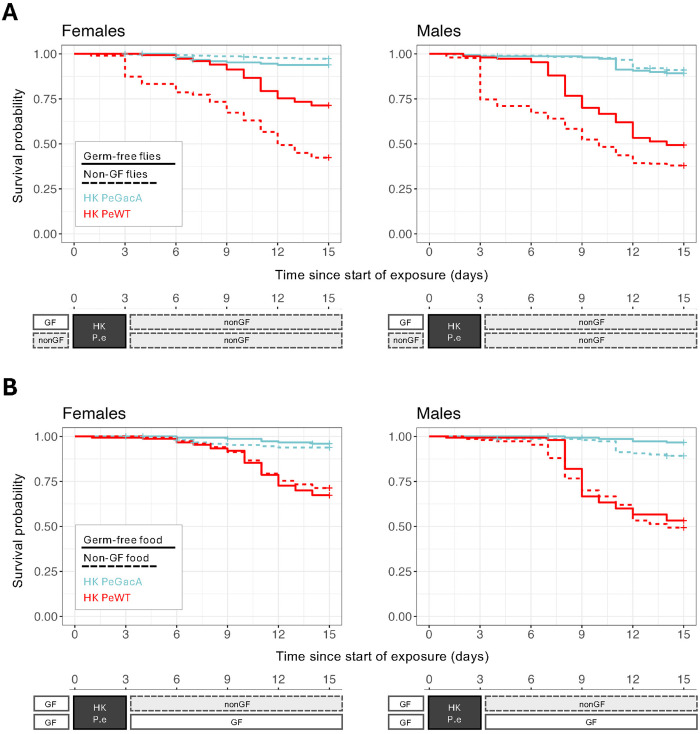
Effects of microbiota on the response to heat-killed *P. entomophila* exposure. Survival curves followed for 14 days for females (left panels) and males (right panels) exposed to heat-killed (HK) *P. entomophila* wildtype (HK PeWT in red) or ΔGacA (HK PeGacA in light blue). A) Survival curves of germ free (solid lines) and non-germ free (dashed lines) flies. Survival differed between germ free and non-free flies when exposed to HK PeWT in both sexes (p < 0.001) but not when exposed to HK PeGacA (p > 0.05). Flies exposed to HK PeGacA show reduced mortality compared to HK PeWT flies, independently of the germ-free treatment (p < 0.05). B) Survival curves of flies transferred to germ free food (solid lines) or normal food (dashed lines). Once again, flies exposed to HK PeWT show higher mortality than those exposed to HK PeGacA (p < 0.001), but no differences were found in flies maintained in germ-free *vs.* normal food (p > 0.05 in both sexes). Note that survival curves for the GF → non-GF condition are repeated in panels A and B for comparison with other treatments.

Further, we tested the role of the gut microbiota in the mortality phenotype observed upon exposure to HK *P. entomophila*. For that, we exposed germ-free and non-germ-free flies to HK PeWT or HK PeGacA and subsequently maintained them on either germ-free or regular food (Fig 5). With this design, we were able to separately assess the role of the microbiota prior to and after exposure to heat-killed bacteria. As before, we found differences in survival between flies exposed to HK PeWT or HK PeGacA (Anova(Cox): χ²(1) = 18.8, p = 1.45e-05) and between sexes (Anova(Cox): χ²(1) = 8.15, p = 0.004) (Fig 5, S1 Table). We also found that the response to HK WT, but not ΔGacA *P. entomophila*, differed between germ free and non-germ free animals (Anova(Cox): χ²(1) = 12.05, p = 5e-04). Mortality was significantly lower in germ-free than in non-germ-free flies exposed to HK PeWT (EMMs(Cox): p < 0.001 in both sexes). This reduction in survival in the presence of microbiota was specific to HK PeWT and not seen with HK PeGacA exposure (Fig 5A, S1 and S8 Tables). Finally, when flies were germ-free at the time of exposure, the type of food they were reared on subsequently, and with this their microbiota status, had no significant effect (Anova(Cox): χ²(1) = 0.91, p = 0.34) (Fig 5B, S1 and S8 Tables).

### 6. Exposure to heat-killed *P. entomophila* triggers an immune response in *D. melanogaster*

We next sought to test how the host immune response reacted to exposure to heat- killed *P. entomophila* [79,80] and measured expression levels of canonical immunity and stress pathway genes in males. For that, we exposed flies to HK PeWT or HK PeGacA and extracted RNA exclusively from adult males, either untreated or at three different time points post-exposure: 8 hours, 72 hours, and 96 hours.

**Fig 6:**
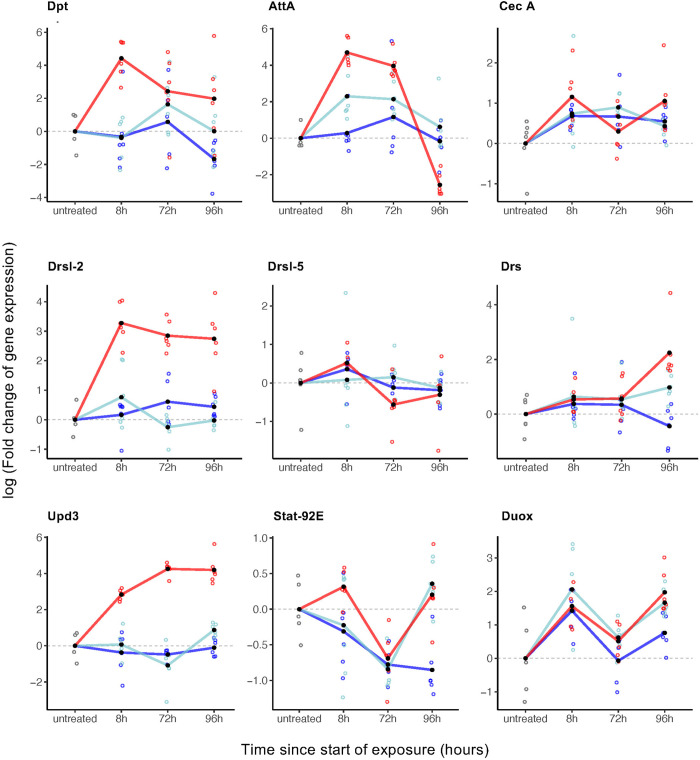
Exposure to HK *P. entomophila* triggers an immune response in *D. melanogaster.* Gene expression (represented as log10 fold change expression normalized to the housekeeping gene *EIF-2*) in cDNA from headless males previously exposed to HK PeWT (red), HK PeGacA (light blue), or PBS control (dark blue). Samples were collected at three different timepoints: 8 hours, 72 hours, and 96 hours after exposure, as well as from untreated individuals on which qPCR was performed for nine canonical immune genes.

We measured the transcriptional profile of canonical antimicrobial peptides (AMPs), reactive oxygen species (ROS), and stress response pathway genes [18,19,91]. We selected genes from the JAK-STAT stress response pathway (i.e., transcription factor *STAT-92E*, its cytokine *Unpaired-3* (*Upd3*)) and two of its gut-specific effectors, *Drosomycin- like 2* (*Drsl-2*) and *Drosomycin-like 5* (*Drsl-5*)). We included genes from both Toll and the IMD pathways (i.e., *Diptericin* (*Dpt*), *Attacin-A* (*AttA*) for IMD, *Drosomycin* (*Drs*) for Toll, and *Cecropin* (*CecA*) for both), as well as from the ROS pathway (i.e., *Dual- oxidase* (*Duox*)) [18,32,83,92] (Fig 6).

Overall, we observed that several genes were upregulated in flies exposed to HK PeWT as compared to those exposed to either PBS or HK PeGacA (Fig 6, S1, S9 Tables). None of the six AMPs showed noticeable expression differences throughout time under the PBS or HK PeGacA treatments (EMMs(lm): p > 0.05). The same unresponsiveness was observed for three AMPs in the HK PeWT treatment, namely *Drsl-5, CecA*, and *Drs* (EMMs(lm) with p > 0.05). In contrast, three other AMPs, *Drsl-2, Dpt*, and *AttA,* were significantly upregulated within the first eight hours of HK PeWT exposure treatments (EMMs(lm) with p < 0.05). Interestingly, *Upd3* showed a similar expression pattern to *Drsl2*, with a consistent up- regulation across all timepoints, exclusively under HK PeWT (EMMs(lm) with p < 0.05 for all timepoints). *STAT-92E*, on the other hand, showed a specific delayed upregulation pattern, detectable at 96h under both HK PeWT (EMMs(lm): est = -1.1 (SE = 0.2), p = 0.03) and HK PeGacA (EMMs(lm): est = -1.2 (SE = 0.2), p = 0.0003) treatments. Finally, *Duox* was constant and unchanged throughout time points and across treatments (Fig 6, S9 Table).

With these results, we conclude that exposure to HK *P. entomophila* triggers stress and immune response pathways in *D. melanogaster*, in a time- and virulence factor-dependent manner.

### 7. The Imd pathway, but not effector genes, is necessary for surviving oral exposure to HK bacteria

**Fig 7:**
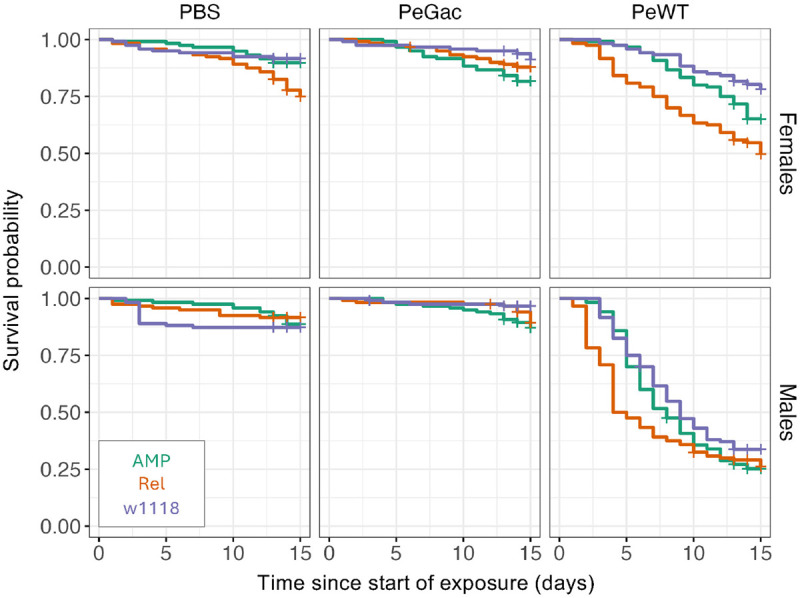
Relish, but not AMP effectors, is involved in mortality caused by feeding on heat-killed PeWT. Survival curves over 14 days for females (top panels) and males (bottom panels), either mutant (Rel^E20^ - orange, ΔAMP - green) or control (background - purple), exposed to heat-killed (HK) *P. entomophila* wildtype (HK PeWT), ΔGacA (HK PeGacA) or PBS. Survival was significantly lower in Rel^E20^ compared to Iso w^1118^ control, when flies were exposed to HK PeWT, for both sexes (p < 0.05) but not when exposed to HK PeGacA or PBS (p > 0.05). There were no differences in survival between ΔAMP flies and control for either HK PeGacA or HK PeWT, in neither sex (p > 0.05). Flies exposed to HK PeGacA show comparable mortality to PBS (p > 0.05) and reduced mortality compared to HK PeWT flies, across all genotypes (p < 0.05).

We found that pathogen virulence is a key factor in the host response to HK *P. entomophila*, which shares multiple similarities with the response to infection by live bacteria [17,62,79]. These parallels led us to test canonical resistance mechanisms and further examine our hypothesis that they play a secondary role in the response to HK bacteria. To this end, we used two mutant lines: a null mutant for the NF-κB transcription factor Relish (Rel^E20^), the key regulator of the IMD pathway (81,94,95), and an AMP mutant lacking 14 different AMPs (ΔAMP) [24,93]. When exposed to HK PeWT, we found no significant differences in survival between the ΔAMP mutant and its control line (Iso w^1118^ background) in either sex (for females (EMMs(Cox): est = 0.5 (SE = 0.38), p = 0.37), for males (EMMs(Cox): est = 0.28 (SE = 0.27), p = 0.55) (Fig 7, S1 and S10 Tables). Conversely, the Rel^E20^ mutant flies, displayed a higher mortality than the control line in both sexes (females: (EMMs(Cox): est = 1.20 (SE = 0.38), p = 4.41e-03); males: (EMMs(Cox): est = 0.72 (SE = 0.26), p = 1.87e-02)) (Fig 7, S10 Table).

## Discussion

In this study, we established an experimental framework to assess disease tolerance in *Drosophila melanogaster* by measuring survival and fecundity following oral exposure to heat-killed *Pseudomonas entomophila* (Figs 1–3). We showed that fitness costs depend on bacterial virulence factors (Figs 2–4), are modulated by the presence of the microbiota (Fig 5), and display marked sexual dimorphism (Figs 1–3, 5, 7). Our results reveal virulence-dependent gut damage and repair responses (Fig 4), a protective role for IMD signalling independent of AMP effectors (Fig 7), and fecundity costs that vary with pathogen virulence (Figs 2–3).

### 1. Establishment of a *P. entomophila* exposure framework to study disease tolerance

We sought to measure disease tolerance in *Drosophila melanogaster* upon oral exposure to *Pseudomonas entomophila* by using an inactivated form of the bacterium, which we hypothesized would minimize the role of host resistance mechanisms. We reasoned that orally exposing *Drosophila* to heat-killed bacteria would trigger its immune response pathways through the detection of antigens that withstood the inactivation treatment. These include cell wall components, such as DAP-type peptidoglycans found in gram-negative bacteria, virulence factors (and toxins in the case of *P. entomophila*), and other pathogen-associated molecular patterns (PAMPS) [40,62,91,94,95].

Bacterial heat inactivation has been successfully used for decades, particularly in the mouse model, to trigger inflammatory responses [96–99], but also in insect species such as in the silkworm *Bombyx mori* [100,101]. *Pseudomonas entomophila* is particularly suitable for this procedure since it is a natural pathogen of *Drosophila* that secretes toxins, inducing strong host responses [75,80,81,88,102].

Feeding flies for 24 hours with live *P. entomophila* negatively affects survival, with observable effects after six hours of continuous feeding [75,81,87]. In contrast, other reported studies [40] show that 24 hours of feeding on heat-killed bacteria does not increase mortality. This informed the establishment of our feeding protocol, which exposes flies to HK bacteria for longer periods, revealing a sustained and repeatable effect on host survival and reproduction (Fig 1).

Due to the different duration of exposures, directly comparing mortality caused by live infection against HK bacteria treatment is not straightforward. By contrasting two *P. entomophila* strains - the wild-type and the non-virulent ΔGacA mutant (Fig 3) [80–82] - we attribute a causal role to virulence factors in driving higher mortality upon oral exposure to HK *P. entomophila*. Within this experimental framework, we show that the presence and activity of virulence factors are pivotal in eliciting disease tolerance responses, as exposure to heat-killed bacteria mirrors the fitness consequences of a live infection (Fig 2, 3, S4).

Finally, the type of bacteria inactivation method, either by heat or with a fixative, yielded similar results (Fig 1A and S3A). However, a somewhat unexpected observation was that bacteria inactivated at 95ºC have a stronger effect on survival than when killed at 55ºC (S3B Table). We expected more denaturation would lead to a stronger reduction of intact PAMPs and, consequently, weaker effects [103,104]. The opposite is observed raising the possibility that at this temperature, more elicitor molecules are presented, a stronger response is deployed, and its deleterious consequences become more acute. Future studies should replicate the experiments presented in this work upon 95ºC bacteria inactivation to unravel the exact effect of heat on virulence factors and toxins after heat-killing *P. entomophila* and compare the magnitude of AMP production and induced tissue damage.

### 2. *P. entomophila* virulence factors activate host immune response

Activation of the *Drosophila* immune response has been associated to shortened life span [105–107], changes in metabolism (most notably in lipid, carbohydrate, and protein metabolism) [108–113] and neurodegeneration [114]. We show in this study that the presence of bacterial virulence factors induces immune activation in adult males, as quantified by the expression of effector genes including AMPs, oxidative stress, and inflammation mediators (Fig 6).

We characterized and compared the expression levels of nine genes at four specific time points: 0h (untreated flies), 8h upon exposure, 72h (end of exposure) and 96h (24h post-exposure), which we anticipated would provide insights onto the recovery phase following exposure.

The production of ROS is a hallmark of live pathogenic infections, notably in *P. entomophila* infections [78,83,115–117]. However, our results fail to show evidence of ROS activity, as there was no upregulation of *Duox* at any of the time points tested. Nonetheless, it is difficult to completely rule out the involvement of oxidative stress in the reported phenotypic effects. Several possible, but not mutually exclusive, explanations could account for the lack of *Duox* transcriptional response. Firstly, the transcriptional unresponsiveness of *Duox* may be attributed to our experimental design, which may not have captured the optimal time window for detecting significant changes in expression. While *Duox* is crucial for the pathogenicity of live *P. entomophila* oral infection, no significant transcriptional differences are observed in fly guts as early as 4h post-infection [83,115]. Secondly, a more direct measurement of the volatile and transient oxidative-stress effector molecules, such as assessing cytochrome c reduction levels, may be necessary to accurately evaluate the role of ROS in the fitness costs induced by ingesting HK bacteria [118]. Thirdly, the unchanged transcriptional profile of *Duox* could be explained by the lack of Uracil production in our heat-killed bacterial context. Uracil has been shown to play a key role in helping the host distinguish between commensals and pathogens, triggering various elimination mechanisms [119,120]. In our setup, heat-killed bacteria likely provide a level of Uracil that is, at best constant, and probably decreasing over time, which may explain the negative result.

In contrast to the *Duox* unresponsiveness, for the other genes studies expression dynamics were, in most cases, different between the wild-type strain, and the avirulent ΔGacA mutant or PBS control treatments, underscoring the link between bacterial virulence and the activation of immune responses [121–123]. Albeit at different stages or routes of infection, previous reports have shown that live *P. entomophila* triggers strong changes in the expression profiles of more than 200 genes including AMPs such as Diptericin, Drosomycin, Cecropins, and Attacins [62,80,83]. Consistently with such previous observations under live infection [79], we found that feeding on HK PeWT bacteria promotes the rapid (within 8 hours) overexpression of three out of six AMPs (*AttA, Dpt,* and *Drsl-2*), while *Drs* showed an upregulation only in the recovery period, i.e., 24 hours after the end of exposure (96h timepoint). This *Drs* delayed response has been observed under oral infection in larvae [80], but not in other contexts, namely in adult systemic infections [62] or in gut microbiota control during metamorphosis [124], where it is upregulated. Together, these data reinforce the context-dependent role of AMPs and their intricate upstream regulation [27,93,125], underscoring the need for a systematic analysis of the underlying regulatory processes that incorporates diverse life-history traits and their ecological relevance.

In this context, as with other epithelial tissues, the three AMPs that respond more strongly are effectors of the IMD and/or JAK/STAT pathways [26,91,94,126]. Accordingly, *Upd3*, another JAK/STAT pathway member, showed sustained upregulation upon HK bacteria exposure. Damage to the gut epithelium has been well documented upon oral live *P. entomophila* infection, with mortality being primarily attributed to the pathogen and the action of its virulence factors, which inhibit host translation and impair cell repair processes [80,115,127]. Such damage is known to activate the JNK and Hippo pathways, which in turn leads to the activation of the JAK-STAT pathway via the cytokine *Upd3* to promote the proliferation of intestinal stem cells, necessary for tissue repair [115,126,128]. Given the above, it is reasonable to speculate that the presence of HK *P. entomophila* and its virulence factors, which cause epithelial damage (Fig 4), closely mimic the effect of live infections, thereby triggering the JAK/STAT-mediated repair process.

The upregulation of specific AMPs in the treatment group exposed to HK PeWT raises the possibility that immunopathology contributes to host mortality, as it is frequently reported as a contributing factor during live infections [18,39]. However, within our experimental framework, we find no indication that this is the case: the AMP expression levels observed here are relatively modest and do not resemble the robust immune responses typically associated with oral infection by live pathogens. Additionally, results from the HK PeGac group, which lacks toxins and virulence factors, showed no significant upregulation of AMPs. This supports our interpretation that neither immunopathology nor resistance mechanisms are likely to be major contributors to the fitness loss observed following HK treatment.

### 3. Heat-killed bacteria induces gut damage and repair in a virulence factor-dependent way

We demonstrated that exposure to a virulent strain of *P. entomophila* induces significantly higher levels of cell death in the gut epithelium than its non-virulent counterpart, the ΔGacA mutant (Fig 4 and S5). This confirmed our hypothesis that oral exposure to heat-killed virulent bacteria induces damage, as previously reported for live infections [83,129]. One important distinction, however, may lie in the duration of the challenge and the timing of damage and repair. In our experimental framework, exposure to HK PeWT leads to a significant increase in cell death, which peaks at the end of the exposure 8- to 10-fold higher than PBS and HK PeGacA treatments (Fig 4C, left panel). This difference diminishes within 48 hours (at the 120 h timepoint), suggesting a temporal limit on the deleterious effects of virulence factor exposure to gut integrity. This observation aligns with the upregulation of the previously described responses involving JAK/STAT activation and concomitant Upd3-mediated increase in expression (Fig 6), shown to promote enterocyte renewal and restoration of epithelium homeostasis [119,128]. This upregulation is initiated within the first hours of exposure, persists throughout the exposure period, and continues for at least 24 hours after the challenge has ended.

In contrast, the expression profile of the JAK/STAT pathway transcription factor *Stat-92E* exhibits more complex dynamics. It is initially upregulated within 8 hours of exposure, then downregulated to homeostatic levels (as defined by PBS treatment) by 72 hours, the peak of exposure time. This is followed by a significant increase 24 hours later, upon transfer to clean food (Fig 6). Evidence suggests that although there is upregulation of damage cytokines (such as *Upd3*) that trigger downstream pathways leading to epithelial repair, this response is blocked due to a translational arrest caused in part by the bacterial toxin Monalysin [76,88,115]. This effect could explain why at 72 hours there is no noticeable difference in cell proliferation between treatments (Fig 4C, right panel). However, the number of proliferating cells at 120 hours, i.e., 48 hours upon release from exposure and presumably the clearance of Monalysin, increases significantly in syntony with the second wave of *Stat-92E* upregulation (Fig 6).

Altogether, these observations support previous evidence of a pathogen-dependent impairment in epithelial repair, which likely plays a role in the death of the fly [83].

### 4. Exposure to HK *P. entomophila* triggers a protective IMD-mediated response independent of AMP effectors

A key premise of our approach to measuring disease tolerance using HK *P. entomophila* is that resistance mechanisms are sufficiently limited to allow the independent assessment of tolerance. Yet, the observed upregulation of a subset of AMPs - key immune effectors involved in epithelial resistance in *Drosophila* [21,129] - raised the question of whether these responses might still contribute to the reduction in fitness observed following HK treatment. We addressed this by performing survival assays in flies mutant either for the IMD pathway transcription factor *Relish* (*Rel*^*E20*^) or for key AMP effectors (ΔAMP flies) including *Dpt* and *AttA*, two of the three AMPs upregulated in our transcriptomic analysis). We found that *Relish* mutants exhibited significantly reduced survival in both sexes, whereas ΔAMP flies showed no significant difference in survival compared to controls following HK *P. entomophila* exposure (Fig 7). These results suggest that AMP effectors are not major contributors to the observed fitness loss and that the increased mortality in *Relish* mutants may reflect additional roles of IMD signalling beyond AMP production. Supporting this interpretation, expression analysis of *Duox*, which encodes the enzyme responsible for ROS production, revealed no significant change in response to HK treatment (Fig 6). Taken together, these findings point to minimal activation of canonical resistance mechanisms - such as AMP production and oxidative stress - under our experimental conditions.

However, in the absence of *Relish*, the IMD pathway designated NF-κB transcription factor, survival to HK *P. entomophila* exposure is impaired (Fig 7). This effect reveals an unsuspected role for Rel in disease tolerance independent of AMP expression and activity. Although AMP expression in the gut is largely attributed to the JAK-STAT pathway [129], the IMD pathway remains central to gut immunity in *D. melanogaster*. It has been shown to trigger oxidative bursts via the NADPH oxidase *Duox* [78,129,130] and to support epithelial regeneration following damage caused by chemicals or pathogens [131–133].

We therefore interpret the increased susceptibility of Relish mutants not as a result of impaired AMP-mediated resistance, but rather as a consequence of disrupted epithelial renewal. This highlights a dual role for Relish in both resistance and tolerance mechanisms. Future studies should aim to disentangle the specific contributions of Relish-mediated pathways in maintaining gut integrity during exposure to HK bacteria.

Future experiments should aim at unveiling the bases for these observations. namely, more thoroughly assess the role of oxidative stress, which may be a key component of the damage caused upon exposure to HK bacteria and explore the genetic underpinnings of the IMD-dependent effect in disease tolerance.

### 5. Microbiota presence lowers the survival of HK-exposed flies

Microbiota profoundly influences host health and physiology, including digestive and immune functions [134–136]. In *Drosophila*, reports show that certain aspects of the microbiota can improve survival and longevity. However, the relationship is complex and context-dependent [137–139]. In this work, mortality following exposure to HK PeWT is exacerbated in the presence of the microbiota, while exposure to HK PeGacA does not show this effect (Fig 5), reiterating the pivotal role of virulence factors in influencing the observed outcomes.

Interestingly, this deleterious effect of the microbiota in survival to HK PeWT feeding depends on when it is present relative to the exposure itself. Flies exposed to HK PeWT with microbiota present throughout the experiment (dashed red lines in Fig 5A), show higher mortality compared to those that were germ-free until being transferred to regular food, post-exposure (solid red lines in Fig 5A). In essence, this difference is driven by whether the microbiota was present or not before and during exposure to HK PeWT. This interpretation is supported by the conditions tested in Fig 5B, where the survival difference disappears when flies are germ-free prior to exposure, regardless of their subsequent microbiota status.

We can propose two hypotheses to accommodate these observations. Possibly, exposure to the wild-type HK bacteria causes dysbiosis, leading to a compound effect of tissue damage and systemic inflammation, which increases host mortality, as described in previous studies [140–142]. Alternatively, or concomitantly, increased mortality in flies with microbiota may result from a shift from localized to systemic response. This would be facilitated by the epithelial damage caused by virulent wild-type bacteria and, possibly, peritrophic matrix deterioration [143] or from signalling to the fat body upon bacterial peptidoglycan detection [144].

Further work is required to test these hypotheses, particularly to understand if and how localized gut damage from ingesting virulent bacteria translates into a systemic response or how the presence of gut microbiota impacts the expression of important immunity effector genes, in the context of the HK PeWT exposure. This work will also prove informative on the distinction between mechanisms of disease tolerance and resistance. Whether microbiome systemic invasion, rather than damage caused by the pathogen itself will allocate the measured effects to resistance or disease tolerance processes, respectively.

### 6. Pervasive sexual dimorphism in survival upon exposure to heat-killed *P. entomophila*

Sexual dimorphism has been reported in several aspects of *Drosophila* physiology, including its immune response [77,145,146]. Several studies have shown that the sexes exhibit profound differences in survival and pathology in response to infections with different kinds of microbes, including bacteria and viruses. Males are known to be more susceptible than females to some acute viral infections [68,147], while the opposite is true in bacterial and fungal infections [145]. These differences in infection responses between sexes are likely due to sex-specific regulation of the different arms of the immune system (e.g., epithelial and systemic immunity) [148,149]. It is important to note, however, that very few studies utilize both sexes for infection-based studies in *Drosophila*. In addition to this, survival outcomes between sexes after infection are heavily dependent on the nature of pathogen and on other external factors like host diet and age [150–152]. Interestingly, male flies consistently show higher mortality in response to HK *P. entomophila* exposure across all our experiments (Figs 1–3, 5 and 7), the opposite to what is typically observed upon live oral bacterial infection [57,145].

The higher mortality observed in males after exposure to HK *P. entomophila* could be explained by different energy allocation to immunity between sexes, as shown by classical examples of immunity/reproduction trade-offs [118,153–158] and suggested by a faster activation and more prolonged immune response reported in *D. melanogaster* males [40].

Another possibility explaining the sexual dimorphism we observe, relies on described sex differences in intestinal physiology [159–161], notably in the proliferation of intestinal stem cells (ISC) [162,163]. Indeed, females repair gut epithelial damage at much faster rates compared to males upon infection or detergent-induced gut damage [159,164]. Recent evidence also shows that epithelial growth factor receptor (EGFR) mutant males display a severe loss of disease tolerance as compared to mutant females [48]. Alternative hypotheses for the observed dimorphic survival outcomes - seemingly more prosaic - might include differences in body size, which can influence metabolic rates, immune function, and overall susceptibility to infection [165]. Larger body size in females, for instance, could confer some level of advantage in resource allocation for immune responses, making them more resilient to the same pathogen.

Another possible explanation for the observed sex differences in mortality is variation in feeding behaviour. During infection with live *P. entomophila*, sex-specific mortality outcomes are typically attributed to females consuming more than males, driven by the higher energetic demands of oogenesis and reproduction [166,167]. However, under our experimental conditions - where flies are exposed to heat-killed bacteria - this feeding pattern may be reversed. Females may be less inclined to feed on HK *P. entomophila* compared to males, potentially contributing to the survival differences observed between sexes. Investigating both genetic and ecological factors that contribute to these sex-specific outcomes will be important for a deeper understanding of disease tolerance and survival strategies across sexes.

### 7. Exposure to heat-killed *P. entomophila* entails fecundity costs

Alongside the extensive characterization discussed above on the effects of exposure to HK *P. entomophila* on survival, we have explored the impact on the other core fitness component, reproduction. Infection with HK bacteria in *Drosophila* is known to result in decreased fecundity [150]. This reduction in fecundity, comparable to that caused by live bacteria, had also been reported and attributed to the cost of activating an immune response [168]. We have replicated these observations with both HK and live bacteria (Figs 1B, 2B, 3B and S4B). Given that exposure to HK bacteria triggers an immune response [62,95], the reduction in fecundity could be explained in part by the immunity-reproduction trade-off previously reported in *Drosophila* and other insect models, as mentioned above [153–157,169]. Immune response and reproduction are both physiologically and energetically demanding processes, requiring the allocation of substantial resources, either from stored reserves or through reallocation [150]. Ultimately, prolonged immune activation may lead to energy depletion, which could be reflected in other fitness traits, such as fertility and fecundity [170].

Additionally, we also observe that the fecundity reduction correlates strongly with bacterial virulence and not with pathogen species *per se*, as our experiments with other non- or mildly-virulent gram-negative bacteria species revealed a higher reproductive output as compared to the HK PeWT-exposed group (Fig 2B). This was confirmed after observing increased fecundity in flies exposed to the avirulent form of the *P. entomophila* under both live and HK conditions (Figs 3B, S4B). This effect is supported by the consistent increase in fecundity observed in females fed any HK bacteria diet. This difference can be attributed to the nutritional quality of the diet whereby the consumption of non-virulent HK bacteria has a greater nutritional value, positively impacting reproductive investment [171]. In contrast, consuming HK bacteria containing virulence factors would impose significant costs on the host, through tissue damage, that the nutritional benefit of the food cannot compensate.

Our results reveal variation in fecundity among flies surviving HK bacterial exposure, suggesting an evolutionary trade-off hinging on disease tolerance. It has been proposed that variation in disease tolerance between individuals (genotypes) may result from a distinct balance between tissue repair and reproduction [11,56,154]. This finding highlights the need for further research exploring the genetic and physiological mechanisms driving disease tolerance for which our experimental framework may contribute. At the same time, because our measurement of fecundity does not distinguish between male and female contributions, it remains unclear whether the sex-specific survival differences we observed also apply to reproductive output. Future studies should address this gap by examining how HK bacterial exposure affects gametogenesis and mating behaviour in both sexes.

This experimental framework has enabled us to integrate fitness traits (i.e., survival and reproduction) to clarify the relationship between host immune response activation and its potential fitness cost. This approach can, not only enhance our understanding of how hosts reduce fitness costs through disease tolerance mechanisms, but also provide insights into how evolution may shape immune strategies against pathogens. While our findings are specific to *P. entomophila* and oral infection, future studies will examine how disease tolerance varies across different pathogens and infection routes, offering deeper insights into host-pathogen interactions and the interplay between disease tolerance and resistance mechanisms.

## Materials and methods

### *Drosophila* stocks and rearing conditions

All experiments, except when indicated, were carried out on an outbred *Drosophila melanogaster* population established and maintained in the lab since 2007 [17,69,172]. The population is maintained in laboratory cages in discrete generations at a census of 1500–2000 individuals, under constant temperature (25°C) and humidity (60–70%) with a 12:12 light-darkness cycle and fed with cornmeal-agar medium, consisting of 4.5% molasses, 7.5% sugar, 7% corn-flower, 2% granulated yeast extract, 1% agar, and 0.25% nipagin, mixed in distilled water. For experiments involving mutant flies, we used the Relish^E20^ (Rel^E20^) [173,174] and the ΔAMP mutant flies previously described elsewhere [24,93]. The ΔAMP line merges distinct deletions removing 14 *Drosophila* AMP genes, namely *Defensin*, *Drosocin*, *Drosomycin*, *Metchnikowin*, two Diptericins (*Dpt A* and *B*), four Cecropins (A1, A2, B and C), and four Attacins (*AttaA*, *B*, *C*, and *D*) in the isogenic (iso w^1118^) background [173]. These lines were generously provided by the Teixeira and Lemaitre labs. All experimental flies were generated from egg lays with controlled density. Three- to five-day-old, mated flies were used for all experiments.

### Bacterial strains and culture

All bacterial strains were cultured in a Luria Bertani (LB) medium with the appropriate antibiotics, when applicable. *Escherichia coli* K-12 (E. coli-K12) was grown at 37°C according to standard protocols. *Pectobacterium carotovora* (Ecc-15), *Pseudomonas entomophila*, and its mutant strain ΔGacA [76,81,82] (both kindly shared by the Lemaitre Lab),as well as *P. putida* (from lab generated stocks were grown at 29°C according to established protocols in the lab [69,172]. Briefly, frozen stocks of bacteria were plated to develop colonies overnight. A single colony was then streaked to make a 5ml starter culture under aseptic conditions and grown in an orbital shaker at 180 rpm for 6 hours. Overnight cultures were then made with a larger volume of LB media using the confluent starter cultures. For live infections, bacteria pellets were collected after centrifugation at 4600rpm for 15 minutes at 4°C and resuspended in sterile LB. For treatment under heat-killed conditions, bacterial pellets were resuspended in sterile PBS and adjusted to the desired concentration (OD600 = 100 for *P. putida*, Ecc-15, as well as for all strains of *P. entomophila*, and OD600 = 200 for *E. coli*).

For the bacteria inactivation with heat, resuspended bacteria with the desired OD were heat-killed for one hour in a water bath at 55°C, for *P. entomophila* and its mutant strains as well as for the two Ecc strains, or at 70°C for *E. coli*. These were the minimal temperatures that did not exhibit any growth (CFUs) upon subsequent plating and overnight incubation at 29°C (S2A Fig). Bacteria inactivation using the fixative paraformaldehyde (PFA) was confirmed in the same manner and prepared by incubating resuspended bacteria pellets in 1% PFA for 30 min. The inactivated bacteria were subsequently washed three times with PBS by centrifugation at 4600 rpm for 10 min at each washing step.

### Fly food preparation and exposure to inactivated bacteria

Liquid fly food was prepared using standard laboratory conditions (as described above) and mixed with the inactivated bacteria in a ratio of 1:1. To account for the texture and viscosity of the food, extra agar powder was added to the mixture (to give an overall 100% agar content after dilution of bacteria) before the addition of the required volume of inactive bacteria solution. In the end, food mixed with agar was dispensed into fly bottles and left to cool down and solidify for at least two hours before use. As a control for the treatment food with bacteria, all the steps were repeated but using sterile PBS instead of inactive bacteria.

### Germ-free *Drosophila* experiment

Egglays were done for one-hour periods in agar plates. Collected eggs were transferred to 2% bleach for 10–15min, followed by a 5 min wash in 70% ETOH for 5min, with two washes in distilled water between each step. We transferred 30–50 eggs to vials with sterile food (autoclaved) and kept there until eclosion. We then generated five replicate vials each containing 10 males and 10 females in either sterile food or regular food in which laboratory flies had been kept on for 24 hours (hence, inoculating the medium with their microbiota). Mortality was followed daily for 15 days with regular flipping to new vials under the same conditions.

### Infection with live bacteria

Oral infection of *D. melanogaster* with *P. entomophila* was performed using the previously established protocols [17,69]. Briefly, starter and overnight cultures were grown using sterile LB media at 29°C as described above. After adjusting the concentration of the pelleted cells to the desired concentration (OD600 = 100) they were diluted 1:1 with 5% sucrose solution, prepared under sterile conditions just before the infection. The resulting mixture was pipetted into filter papers and placed inside food vials to which groups of 10 single-sex flies were transferred, to feed for 24 hours at 25°C. Later, they were moved to clean vials with normal food where survival was measured for six days. As a control to the infection experiment, flies were fed with the 5% sucrose solution without the bacteria.

### Phenotyping survival and fecundity

Flies were mated and aged three to five days for all experiments. Bacteria exposure treatment was done with 15 females and 15 males per vial except for experiments with bacterial mutants, where we used 10 males and 10 females per vial. Survival measurements began at the start of the treatment and lasted 21 days or 14 days, depending on the experiment (see Results). Flies that escaped or sustained accidental injury during transfer were censored from the analysis, as well as flies that died within the first hour of the experiment.

Fecundity was measured by transferring adults daily to new vials and counting the number of pupae produced each day (S1 Fig). Melanized pupae and third-instar larvae were excluded from the count. Fecundity was calculated by normalizing the number of pupae to the daily count, as melanization typically indicates death. Fecundity was calculated by normalizing the number of pupae to the daily count of alive females, providing an estimate of daily fecundity per female. Lifetime fecundity per female was determined by summing these daily values over the specified period.

### Gut sample staining and quantification

Intestines of three-to-five-day-old males exposed to the different HK bacteria treatments at specific time points were dissected in cold PBS and immediately fixed in 4% formaldehyde for 30 mins. Fixation followed three 15min washes with PBS- T (0.1% Triton X-100/PBS) and blocking for 1 hour at room temperature with 2% BSA (2% BSA/0.1% Triton X-100/PBS). Guts were then incubated overnight at 4°C with primary antibodies diluted in blocking solution (1:200 rabbit anti-Dcp1, Cell Signaling #9578; 1:500 mouse anti-PH3, Cell Signaling #9706) followed by three washes in PBS-T and a 2-hour room temperature incubation with secondary antibodies (anti- rabbit Alexa Fluor 568; anti-mouse Alex Fluor 488) and (1:1000) DAPI. After three washes, samples were mounted in 90% glycerol. Images were acquired on a Leica TCS SP5 confocal microscope, using a 20x/0.7 objective and 405, 488, and 532 laser lines to excite DAPI, Alexa488, and Alexa568, respectively. Images of ~6 male midguts were used to manually quantify fluorescent cells for Dcp-1 (a marker for apoptosis, [175] and PH3 (a marker for proliferation; [176]) for equivalent portions of posterior midgut regions.

### RNA extractions and RT-qPCR

For all RNA extractions, flies were exposed to the protocol described above and, at different time points (0h or untreated, 8 hours, 72 hours, and 96 hours post-exposure) three headless males per treatment and time point were pooled and, subsequently, manually homogenized using a sterile pestle in 500 µL of Trizol. RNA extractions were performed using a phenol-chloroform protocol from which 1 µg of RNA was used per pooled sample replicate for cDNA synthesis. After precipitation, we performed a DNase I treatment (RQ1 RNASE-FREE DNASE 1* from Promega), followed by reverse transcription using the Thermo Scientific RevertAid H Minus cDNA kit. cDNA was diluted 1:5 for the qPCR. For quantification of gene expression, qPCRs were performed using Green Master Mix (Thermo Scientific) and reactions ran on 96-well plates (Applied Biosystems). The PCR conditions used in all experiments were: initial denaturation/ enzyme activation, 95ºC for 10 min; followed by 45 cycles of denaturation, 95ºC for 10 sec; annealing, 60ºC for 10 sec; extension, 72ºC for 30 sec. Sequences of primers used for qPCRs are shown in S11 Table with *EIF2* as a reference housekeeping gene.

Gene expression analysis was performed using relative quantification (ΔΔCt), using the average of the technical replicates for each candidate gene, subtracting the average of the Ct values of the respective sample’s housekeeping gene (*EIF2*) (ΔCt) and normalizing this value to the ΔCt of the respective reference condition.

### Statistical analysis

All statistical analyses were conducted in R v4.2.1 [177] using the following R packages: *tidyverse* [178] for data manipulation, *ggplot2* [179] for visualization, *lme4* [180] and *lmerTest* [181] for linear mixed-effects models, *glmmTMB* [182] and *pscl* [183] for zero-inflated models, and *emmeans* [184] for post- hoc multiple comparisons.

Survival data were analysed using Cox proportional hazards models, followed by Type III ANOVA to assess the effects of fixed factors. Fecundity data (measured as the sum of daily fecundity) and data on the number of Dcp-1 or PH3 positive cells were analysed using zero-inflated negative binomial models. Exceptionally, for the experiment in Fig 1, fecundity data was measured as the number of pupae by the end of the experiment, and this data was analysed using linear mixed-effects models. For both survival and fecundity data, post-hoc pairwise comparisons among treatment groups were conducted using estimated marginal means, with significance set at p < 0.05. For all experiments, models included relevant fixed factors (e.g., Treatment, Sex, Days of Exposure, Bacterial species, Bacterial strain), while accounting for random effects from bottles or vials where applicable. Full model details and outputs are provided in the supplementary tables.

## Supporting information

S1 Fig Experimental setup for measuring survival and reproductive output of flies using heat-killed *P. entomophila.* Bacteria were grown under standard conditions (29°C at 180 RPM) and the OD was adjusted accordingly (OD600 = 100), followed by a water bath incubation at 55°C for one hour, and freezing until later usage. Three-to-five-day-old flies were either exposed to fly food mixed 1:1 with HK *P. entomophila* or with PBS (control food) for three days and monitored daily for survival. After three days, flies were placed on normal food and flipped daily into new vials for at least 12 days. Survival and pupal counts were measured in each of the vials where flies were maintained. Red circles indicate the period of exposure to food mixed with HK *P. entomophila*, while blue circles indicate periods where flies were kept on normal food. Images from https://creazilla.com/media/clipart/3168246/test.
(TIF)

S2 Fig Inactivation of *P. entomophila* by heat-killing and its effects on *Drosophila* survival across sexes and exposure durations. **A)** *P. entomophila* cultures were prepared following standard protocols, resuspended in PBS to OD₆₀₀ = 100, and streaked onto LB+agar plates containing 100 µg/ml rifampicin. The presence of colonies after 24 hours at 29 °C (left) confirms bacterial viability. No colonies were observed after heat-killing the culture at 55 °C for 1 hour and plating on rifampicin-containing (middle) or standard LB+agar (right), confirming successful inactivation. **B)** Hazard ratios by sex (females in magenta and males in grey) and exposure duration (X axis). Ratios represent survival in response to HK *P. entomophila* relative to the PBS control. Note that high hazard ratios with wide confidence intervals likely result from small sample sizes and pronounced mortality differences between treatments at specific time points.
(TIF)

S3 Fig Alternative inactivation methods confirm effect on survival. Survival over 14 days of female (left plots) and male (right plots) flies exposed to food containing wild-type *P. entomophila* inactivated with different methods. **A)** Survival upon exposure to food containing *P. entomophila* heat-killed at 55 ºC (HK PeWT - red) or fixed with paraformaldehyde (PFA) (PFA PeWT - orange), containing PFA alone (PFA control - grey) or PBS (PBS control - blue). There is no significant difference between survival measurements in the group fed with HK *P. entomophila* and that of PFA control (p > 0.05), but both are different from control treatments (p < 0.001). **B)** Survival upon exposure to food with HK *P. entomophila* at 55 ºC (HK PeWT-55 ºC - red) or 95 ºC (HK PeWT-95 ºC - brown) or PBS control food (PBS - blue). There were differences in survival between inactivation temperatures in females (p < 0.001), but not in males (p > 0.05).
(TIF)

S4 Fig Oral infection with wildtype *P. entomophila* leads to increased mortality and reduced fecundity in *D. melanogaster.* Survival and fecundity in adult flies orally infected with either wild-type *P. entomophila* (PeWT - red), the avirulent *P. entomophila* Δ*GacA* mutant (PeGacA - light blue), or 5% sucrose control solution (Sucrose - grey). **A)** Survival curves of females (left panel) and males (right panel) over 6 days show a significant difference in survival between the PeWT- and the GacA-infected or the sucrose individuals(p < 0.001 in both cases). **B)** Fecundity (measured as cumulative daily pupal counts; see Material and Methods) shows that flies infected with wildtype PeWT are significantly less fecund than the PeGacA-infected and the sucrose group (p < 0.001 in both cases). PeGacA-infected flies also showed a significantly higher reproductive output than flies from the Sucrose group. Differences between groups were estimated by post hoc comparisons (Tukey’s honest significant differences) and are indicated by different letters in each plot (p < 0.05).
(TIF)

S5 Fig Feeding on heat-killed *P. entomophila* induces apoptosis and mitotic cell division in gut epithelial tissues. Immunofluorescent staining using antibodies against Drosophila caspase 1 (Dcp-1) in red, Phospho-H3 (PH3) in green and merged with DAPI in blue. Images are taken from the posterior midgut of males untreated **(A)** 72h **(B)** and 120h **(C),** after treatment with heat-killed wildtype (HK PeWT) or mutant ΔGacA (HK PeGacA) *P. entomophila*. Images are displayed per channel and merged in the final column. These images are representative of at least six replicates per treatment and time points.
(TIF)

S1 Table ANOVA Results. Table shows the results of the ANOVA models for each experiment and trait, including the fitted models, degrees of freedom (Df), chi-square values (Chisq), and the associated p-values (Pr(>Chisq) for each variable in the model. Non-significant differences (p > 0.05) are indicated by grey p-values.
(XLSX)

S2 Table Results of post-hoc comparisons for experiment of the days of exposure. Table shows the results of multiple pairwise comparisons using the emmeans function for survival and fecundity traits in the experiment testing different days of exposure. It includes details of the fitted models, the type of comparison performed, the contrast groups, and the corresponding estimated effect size (estimate), standard error (SE), degrees of freedom (df), z-ratio, and p-value for each comparison. Non-significant differences (p > 0.05) are indicated by grey p-values.
(XLSX)

S3 Table Results of posthoc comparisons for experiment of the inactivation methods. Table shows the results of multiple pairwise comparisons using the emmeans function for survival in the experiment testing different inactivation methods. It includes details of the fitted models, the type of comparison performed, the contrasted groups, and the corresponding estimated effect size (estimate), standard error (SE), degrees of freedom (df), z-ratio, and p-value for each comparison. Non-significant differences (p > 0.05) are indicated by grey p-values.
(XLSX)

S4 Table Results of posthoc comparisons for experiment of the bacterial species. Table shows the results of multiple pairwise comparisons using the emmeans function for survival and fecundity in the experiment testing the effects of different bacterial species. It includes details of the fitted models, the type of comparison performed, the contrasted groups, and the corresponding estimated effect size (estimate), standard error (SE), degrees of freedom (df), z-ratio, and p-value for each comparison. Non-significant differences (p > 0.05) are indicated by grey p-values.
(XLSX)

S5 Table Results of posthoc comparisons for experiment of the bacterial mutants. Table shows the results of multiple pairwise comparisons using the emmeans function for survival and fecundity in the experiment testing the effects of different bacterial mutants. It includes details of the fitted models, the type of comparison performed, the contrasted groups, and the corresponding estimated effect size (estimate), standard error (SE), degrees of freedom (df), z-ratio, and p-value for each comparison. Non-significant differences (p > 0.05) are indicated by grey p-values.
(XLSX)

S6 Table Results of posthoc comparisons for experiment of the live infection. Table shows the results of multiple pairwise comparisons using the emmeans function for survival and fecundity in the experiment testing the effects of a live infection. It includes details of the fitted models, the type of comparison performed, the contrasted groups, and the corresponding estimated effect size (estimate), standard error (SE), degrees of freedom (df), z-ratio, and p-value for each comparison. Non-significant differences (p > 0.05) are indicated by grey p-values.
(XLSX)

S7 Table Results of posthoc comparisons for experiment of the gut immunostaining. Table shows the results of multiple pairwise comparisons using the emmeans function for the count data of the experiment of gut immunostainings. It includes details of the fitted models, the contrasted groups, and the corresponding estimated effect size (estimate), standard error (SE), degrees of freedom (df), z-ratio, and p-value for each comparison. Non-significant differences (p > 0.05) are indicated by grey p-values.
(XLSX)

S8 Table Results of posthoc comparisons for the germ-free experiment. Table shows the results of multiple pairwise comparisons using the emmeans function for survival traits in the germ-free experiment. It includes details of the fitted models, the contrasted between treatment groups, and the corresponding estimated effect size (estimate), standard error (SE), degrees of freedom (df), z-ratio, and p-value for each comparison. Non-significant differences (p > 0.05) are indicated by grey p-values.
(XLSX)

S9 Table Results of posthoc comparisons for the qPCR gene expression experiment. Table shows the results of multiple pairwise comparisons using the emmeans function for gene expression data. It includes details of the fitted models, the contrasts between treatment groups, and the corresponding estimated effect size (estimate), standard error (SE), degrees of freedom (df), z-ratio, and p-value for each comparison. Non-significant differences (p > 0.05) are indicated by grey p-values.
(XLSX)

S10 Table Results of posthoc comparisons for the Fly mutants experiment. Table shows the results of multiple pairwise comparisons using the emmeans function for different fly lines. It includes details of the fitted models, the contrasts between treatment groups, and the corresponding estimated effect size (estimate), standard error (SE), degrees of freedom (df), z-ratio, and p-value for each comparison. Non-significant differences (p > 0.05) are indicated by grey p-values.
(XLSX)

S11 Table Primer list and sequences. (XLSX)

S1 File R code - Exposure time. Survival and fecundity analyses upon exposure to HK *P. entomophila* under varying exposure times.
(R)

S2 File R code - Inactivation methods. Survival analysis comparing two bacterial inactivation methods (PFA and 95 °C). Includes Cox mixed-effects models, contrasts, and survival plots.
(R)

S3 File R code - Bacterial species. Survival and fecundity analyses for HK bacterial exposure for different species. Includes Cox mixed-effects models, contrasts, and survival plots.
(R)

S4 File R code - *Pseudomonas entomophila* strains. Survival and fecundity analyses for *Drosophila* exposed to different bacterial strains.
(R)

S5 File R code - Germ-Free. Survival analysis of *Drosophila* exposed to HK *P. entomophila* under germ-free or undisturbed microbiota (both before and after exposure).
(R)

S6 File R code - qPCR Analysis. Immune gene expression upon exposure to HK *P. entomophila*. Analysis of log-fold change in gene expression after bacterial treatments using qPCR data.
(R)

S7 File R code - Staining. Analyses of Dcp1 (apoptosis) and Ph3 (proliferation) gut staining upon exposure to HK *P. entomophila*.
(R)

S8 File R code - Fly Mutants. Survival analysis upon exposure to HK *P. entomophila* of *D. melanogaster* mutant stains (Relish^E20^ and ΔAMP). Includes Cox mixed-effects models of *Drosophila* survival across genotypes, treatments, and sexes.
(R)

S9 File Raw Data Table. Excel file containing the raw data for the 12 datasets included in the manuscript.
(XLSX)
