# Supplementary figures and images for: Pathogen-induced damage in Drosophila: Uncoupling disease tolerance from resistance
Source: PLoS Pathog. 2025 Sep 19;21(9):e1013482. doi: 10.1371/journal.ppat.1013482 (PMC12463329; doi:10.1371/journal.ppat.1013482)

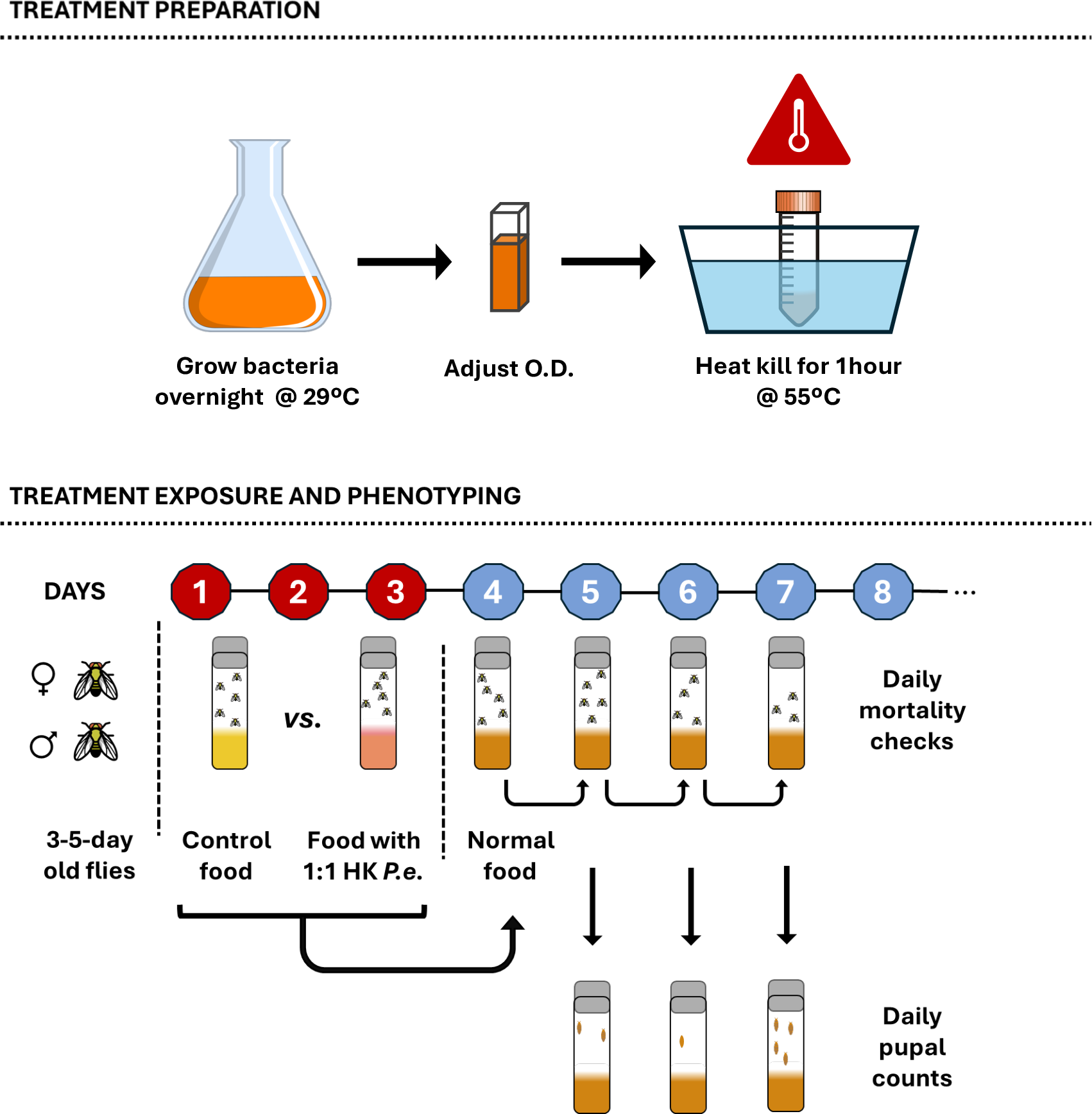

Supplement: S1 Fig — Bacteria were grown under standard conditions (29°C at 180 RPM) and the OD was adjusted accordingly (OD600 = 100), followed by a water bath incubation at 55°C for one hour, and freezing until later usage. Three-to-five-day-old flies were either exposed to fly food mixed 1:1 with HK P. entomophila or with PBS (control food) for three days and monitored daily for survival. After three days, flies were placed on normal food and flipped daily into new vials for at least 12 days. Survival and pupal counts were measured in each of the vials where flies were maintained. Red circles indicate the period of exposure to food mixed with HK P. entomophila, while blue circles indicate periods where flies were kept on normal food. Images from https://creazilla.com/media/clipart/3168246/test. (TIF) [file ppat.1013482.s001.tif]

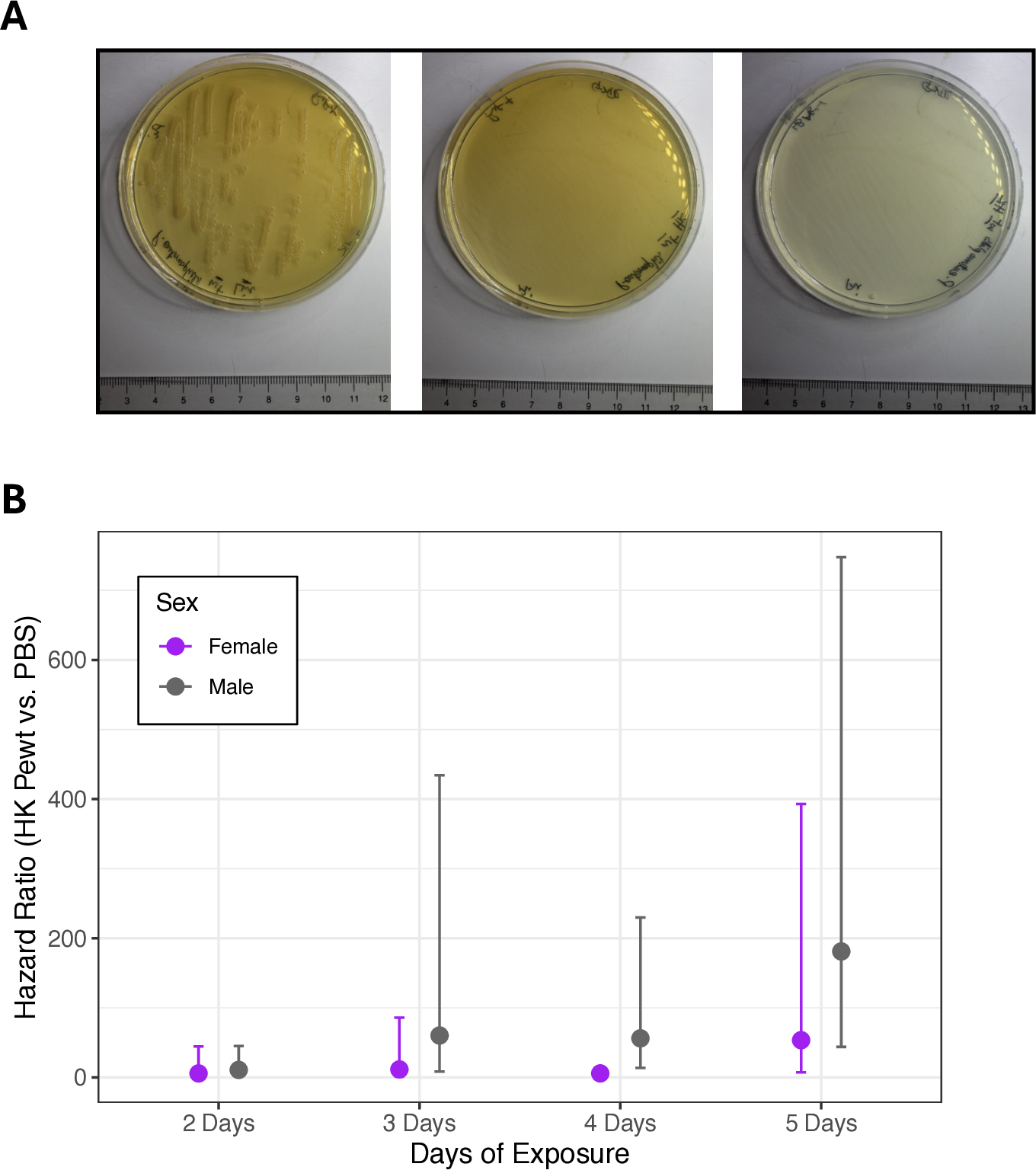

Supplement: S2 Fig — A) P. entomophila cultures were prepared following standard protocols, resuspended in PBS to OD₆₀₀ = 100, and streaked onto LB+agar plates containing 100 µg/ml rifampicin. The presence of colonies after 24 hours at 29 °C (left) confirms bacterial viability. No colonies were observed after heat-killing the culture at 55 °C for 1 hour and plating on rifampicin-containing (middle) or standard LB+agar (right), confirming successful inactivation. B) Hazard ratios by sex (females in magenta and males in grey) and exposure duration (X axis). Ratios represent survival in response to HK P. entomophila relative to the PBS control. Note that high hazard ratios with wide confidence intervals likely result from small sample sizes and pronounced mortality differences between treatments at specific time points. (TIF) [file ppat.1013482.s002.tif]

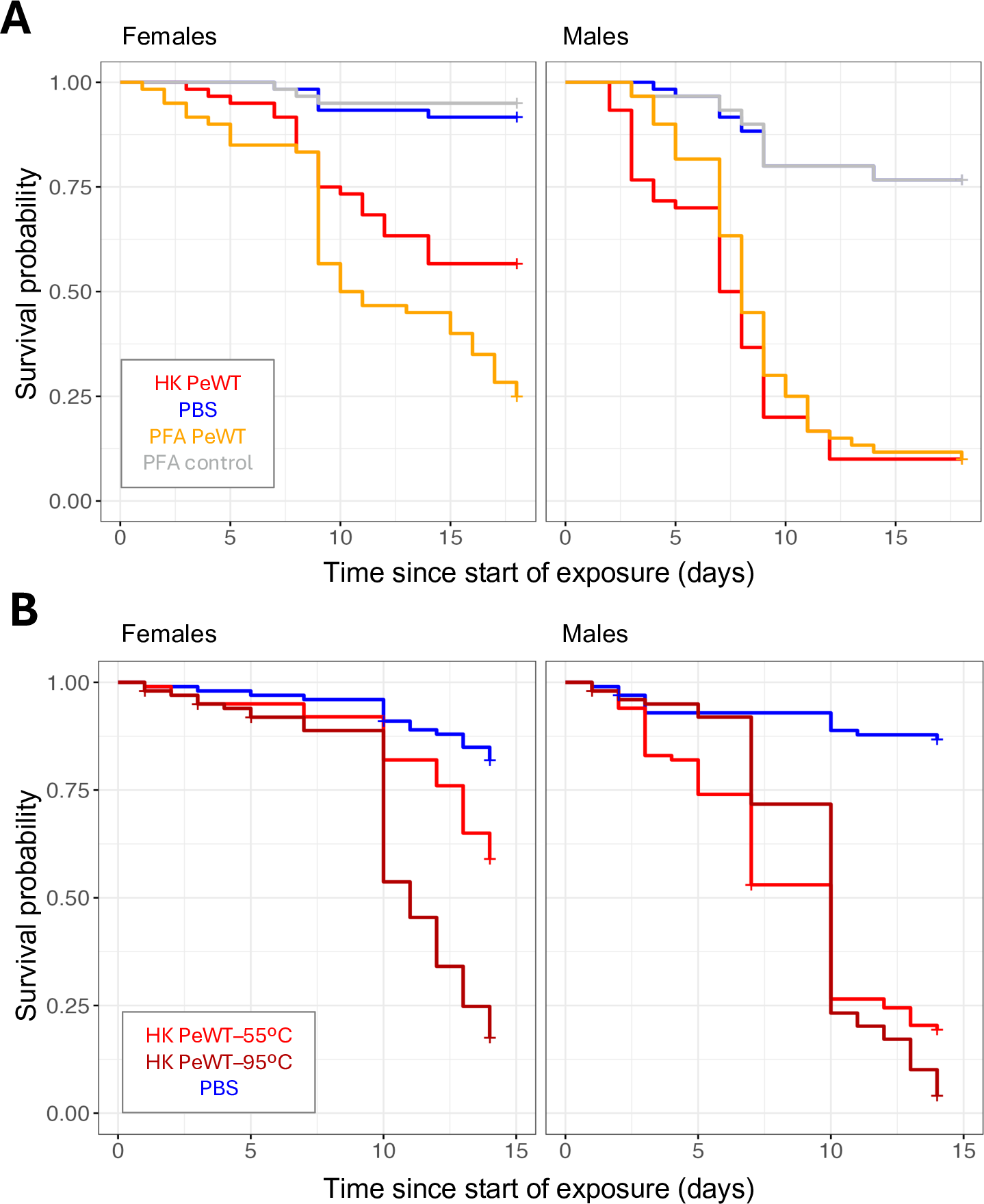

Supplement: S3 Fig — Survival over 14 days of female (left plots) and male (right plots) flies exposed to food containing wild-type P. entomophila inactivated with different methods. A) Survival upon exposure to food containing P. entomophila heat-killed at 55 ºC (HK PeWT - red) or fixed with paraformaldehyde (PFA) (PFA PeWT - orange), containing PFA alone (PFA control - grey) or PBS (PBS control - blue). There is no significant difference between survival measurements in the group fed with HK P. entomophila and that of PFA control (p > 0.05), but both are different from control treatments (p < 0.001). B) Survival upon exposure to food with HK P. entomophila at 55 ºC (HK PeWT-55 ºC - red) or 95 ºC (HK PeWT-95 ºC - brown) or PBS control food (PBS - blue). There were differences in survival between inactivation temperatures in females (p < 0.001), but not in males (p > 0.05). (TIF) [file ppat.1013482.s003.tif]

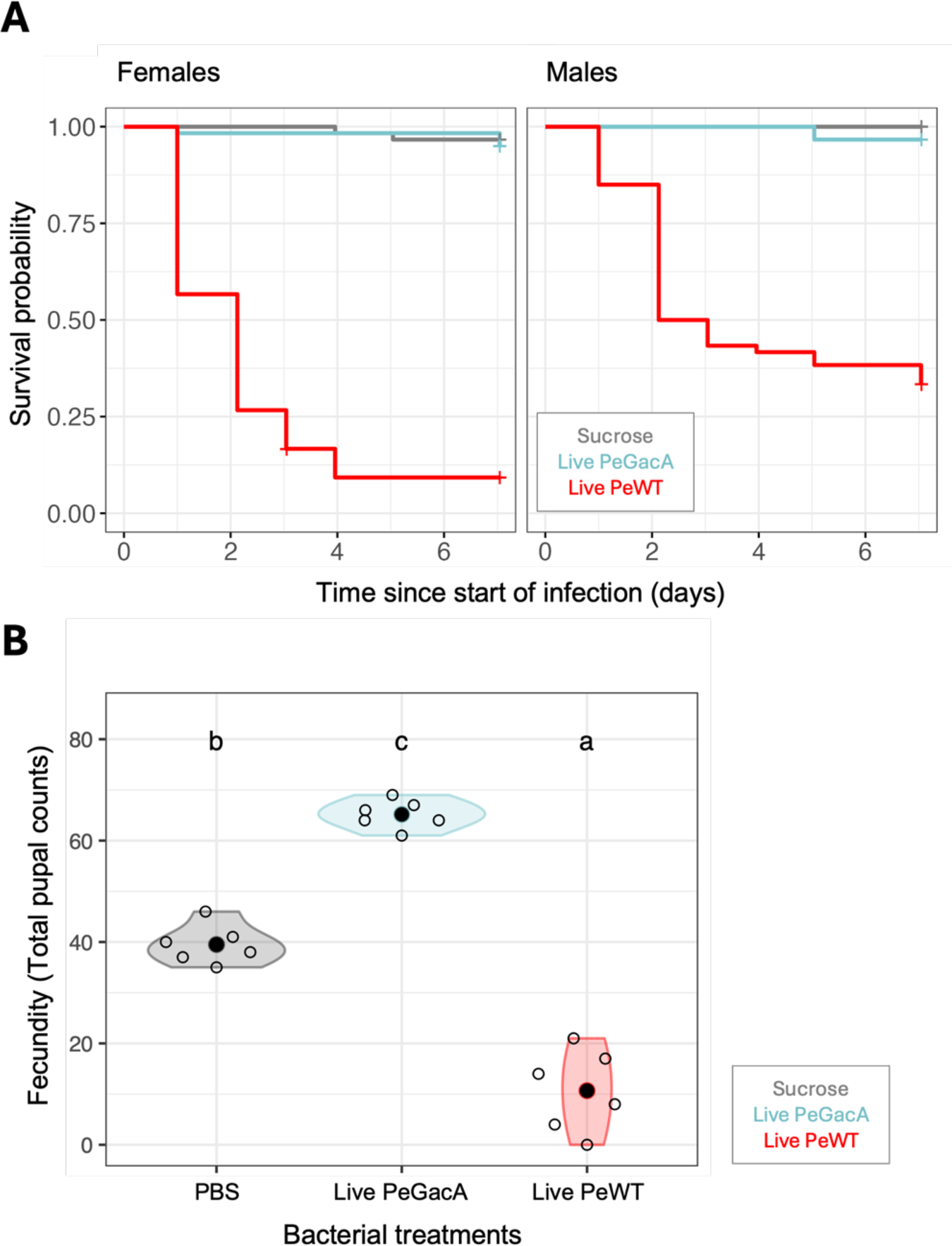

Supplement: S4 Fig — Survival and fecundity in adult flies orally infected with either wild-type P. entomophila (PeWT - red), the avirulent P. entomophila ΔGacA mutant (PeGacA - light blue), or 5% sucrose control solution (Sucrose - grey). A) Survival curves of females (left panel) and males (right panel) over 6 days show a significant difference in survival between the PeWT- and the GacA-infected or the sucrose individuals(p < 0.001 in both cases). B) Fecundity (measured as cumulative daily pupal counts; see Material and Methods) shows that flies infected with wildtype PeWT are significantly less fecund than the PeGacA-infected and the sucrose group (p < 0.001 in both cases). PeGacA-infected flies also showed a significantly higher reproductive output than flies from the Sucrose group. Differences between groups were estimated by post hoc comparisons (Tukey’s honest significant differences) and are indicated by different letters in each plot (p < 0.05). (TIF) [file ppat.1013482.s004.tif]

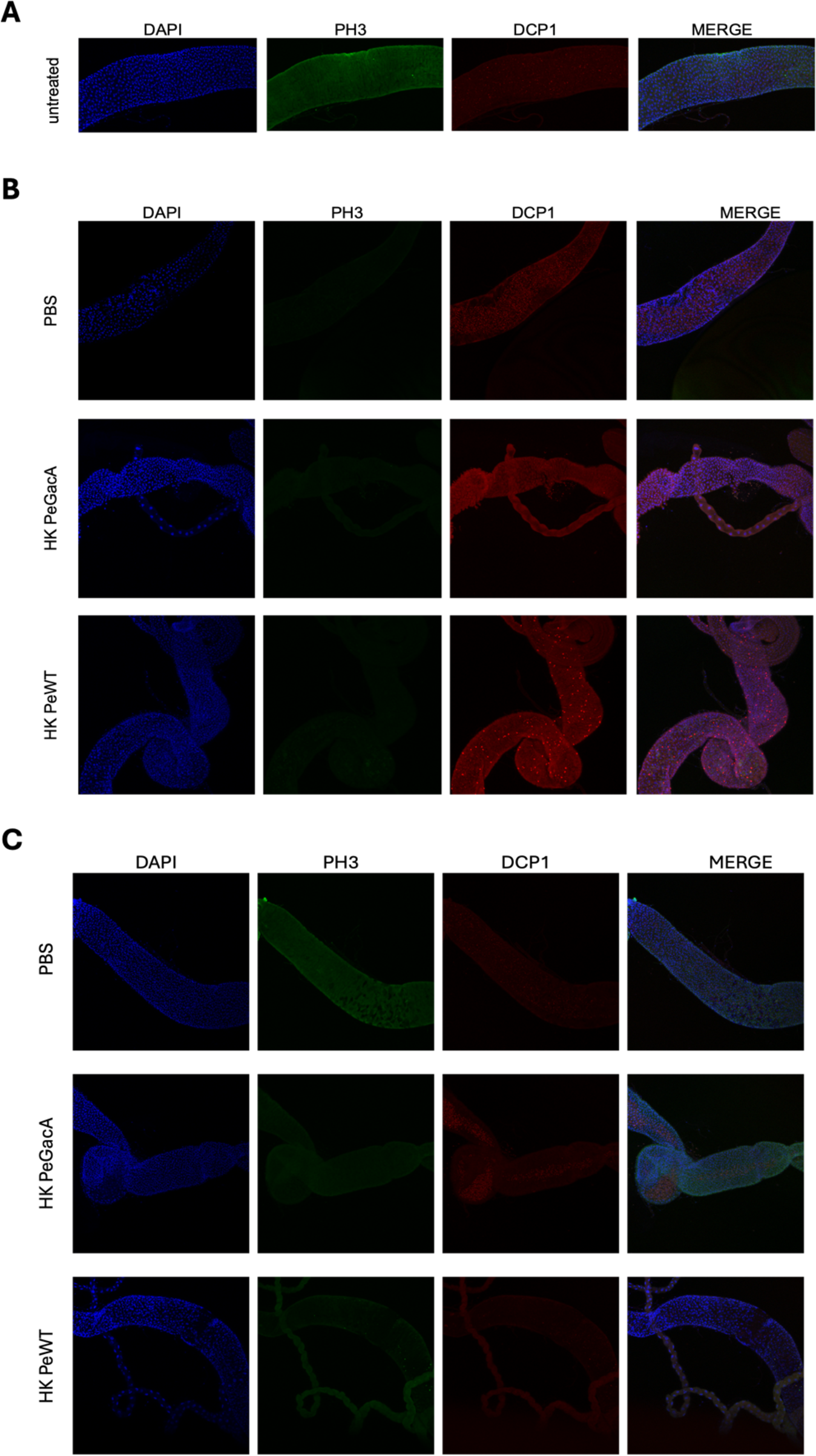

Supplement: S5 Fig — Immunofluorescent staining using antibodies against Drosophila caspase 1 (Dcp-1) in red, Phospho-H3 (PH3) in green and merged with DAPI in blue. Images are taken from the posterior midgut of males untreated (A) 72h (B) and 120h (C), after treatment with heat-killed wildtype (HK PeWT) or mutant ΔGacA (HK PeGacA) P. entomophila. Images are displayed per channel and merged in the final column. These images are representative of at least six replicates per treatment and time points. (TIF) [file ppat.1013482.s005.tif]
